# EZH2 at the epigenetic–immune interface: a dual-axis framework for tumor immune evasion and precision combination immunotherapy

**DOI:** 10.3389/fimmu.2026.1909464

**Published:** 2026-10-26

**Authors:** Yang Bai, Huimin Chen, Ye Gu

**Affiliations:** Department of Pediatric Surgery, The Second Hospital of Jilin University, Changchun, China

**Keywords:** adoptive cell therapies, CAR T-cell therapy, EZH2, immune checkpoint blockade, tumor-intrinsic invisibility

## Abstract

Despite the transformative activity of immune checkpoint blockade (ICB) and adoptive cellular therapies in selected malignancies, durable responses remain limited in many solid tumors, in part because tumor-intrinsic and microenvironmental epigenetic programs can restrict immune recognition and antitumor effector function. Enhancer of zeste homolog 2 (EZH2), a catalytic subunit of polycomb repressive complex 2 (PRC2), contributes to this regulation through canonical H3K27 trimethylation-dependent repression, as well as context-dependent functions that extend beyond a simple model of uniform immune suppression. We organize the available evidence into a dual-axis framework in which tumor-intrinsic EZH2/PRC2 activity can regulate major histocompatibility complex class I and II antigen presentation, interferon and viral-mimicry programs, programmed death-ligand 1 (PD-L1), and T helper 1 (TH1)-type chemokines, whereas tumor-extrinsic EZH2 activity influences macrophages, regulatory T cells, CD8^+^ T cells, and natural killer (NK) cells. Importantly, these effects are not uniformly immunostimulatory: systemic or prolonged EZH2 perturbation has also been associated with myeloid-derived suppressor cell (MDSC) expansion in selected preclinical settings. Early-phase human studies established the feasibility, but not the incremental clinical benefit, of combining tazemetostat with programmed cell death protein 1 (PD-1)/PD-L1 blockade or chimeric antigen receptor (CAR) T-cell therapy; its subsequent withdrawal after a hematologic safety signal leaves further clinical testing of this strategy to other EZH2- or EZH1/EZH2-directed agents. These observations support mechanism-matched, biomarker-informed, compartment-aware, and temporally controlled EZH2 modulation rather than indiscriminate systemic EZH2 inhibition as the more appropriate framework for future immunotherapy combinations.

## 1 Introduction

Cancer immunotherapy has advanced substantially in recent years, particularly through immune checkpoint blockade (ICB) (1). Immune checkpoint inhibitors (ICIs) block regulatory pathways that suppress T cell activity and immune responses, leading to robust immune system (2–5). Mechanistically, cancer cells can exploit these immune checkpoints to evade host immune surveillance, offering a potential strategy for cancer immunotherapy (6–11). Despite the significant clinical benefits observed in some patients, with notable tumor regression in response to ICIs, a substantial proportion of patients either do not respond to these treatments or exhibit low response rates (12–15). Some patients experience innate resistance or develop acquired resistance over time, diminishing the effectiveness of ICIs (16–18). This therapeutic ceiling has redirected attention toward cancer-intrinsic immune-evasion mechanisms upstream of checkpoint biology, among which epigenetic dysregulation has emerged as a particularly tractable vulnerability, being reversible, druggable, and capable of restoring tumor immunogenicity in a coordinated, multi-gene fashion (19, 20). Loss of major histocompatibility complex (MHC) class I antigen presentation can contribute to resistance to immune checkpoint inhibition and, in selected tumor contexts, can be maintained through reversible epigenetic repression rather than through irreversible genetic loss (19, 20). Analogous epigenetic mechanisms can regulate T-cell-recruiting chemokines and immunosuppressive cellular programs within the tumor microenvironment (19, 21).

Within this landscape, enhancer of zeste homolog 2 (EZH2), a catalytic methyltransferase of polycomb repressive complex 2 (PRC2), has crystallized as a master epigenetic node coupling oncogenic transcription to immune escape (22, 23). Critically, EZH2’s role in tumor immunity is *bidirectional*. *Tumor-intrinsically*, EZH2 silences MHC-I/II loci (24, 25), T-cell-recruiting chemokines such as CXCL9/CXCL10 (21), CCL5 (26), and immunogenic transcriptional programs (23). Tumor-extrinsically, EZH2 has been implicated in the differentiation or functional state of CD8^+^ T cells, regulatory T cells, NK cells, and myeloid populations within the tumor microenvironment (TME) (27, 28). This dual-axis architecture raises the possibility that EZH2 inhibition could concurrently influence antigen presentation, T-cell trafficking, and the composition of the suppressive TME. As detailed in Sections 5.5 and 8.1, however, these effects are not uniformly favorable, and tumor-directed benefit can be offset by opposing changes in myeloid and activated effector T-cell compartments (29–31).

The selective oral EZH2 catalytic inhibitor tazemetostat (EPZ-6438) received accelerated approval from the U.S. FDA in 2020 for epithelioid sarcoma and for defined relapsed/refractory follicular lymphoma indications (32, 33). The dual EZH1/EZH2 inhibitor valemetostat was subsequently approved in Japan in 2022 for relapsed/refractory adult T-cell leukemia/lymphoma and in 2024 for relapsed/refractory peripheral T-cell lymphoma (34), and the selective EZH2 inhibitor zeprumetostat (SHR2554) received conditional approval in China in 2025 for relapsed/refractory peripheral T-cell lymphoma. Building on these approvals, clinical studies have evaluated EZH2- and EZH1/EZH2-directed agents in combination with checkpoint blockade, CAR T-cell therapy, and T-cell-engaging approach (28, 35). The subsequent clinical development and current regulatory status of EZH2- and EZH1/EZH2-directed therapies are discussed in Section 7. Outcomes nonetheless remain context-dependent, with EZH2 inhibition occasionally impairing effector immunity, a duality that defines the central tension of the field (27, 28). This review provides an integrated, mechanism-anchored synthesis of EZH2 in cancer immunology, traversing the structural foundations of EZH2/PRC2, clinical biomarkers, tumor-intrinsic immune evasion, TME reprogramming, combination immunotherapy strategies, resistance mechanisms, and the next-generation therapeutic outlook.

## 2 EZH2 and PRC2: structural, catalytic, non-canonical foundations, and epigenetic-network foundations

### 2.1 PRC2 architecture and canonical and non-canonical EZH2 functions

EZH2 is an evolutionarily conserved histone methyltransferase that operates as the catalytic subunit of polycomb repressive complex 2 (PRC2) and sequentially mono-, di-, and trimethylates lysine 27 of histone H3 (H3K27me1/me2/me3) (36, 37). Although H3K27me2 is the most abundant of these marks genome-wide, H3K27me3 is the modification most directly associated with stable Polycomb-mediated transcriptional repression and is a defining hallmark of facultative heterochromatin (36, 38). The human EZH2 gene maps to chromosome 7q36.1, within the region historically reported as 7q35, and generates multiple alternatively spliced transcripts; the canonical isoform encodes a 746-amino-acid protein organized into five functional regions: the EED-interaction domain (EID), Domain I, Domain II, a cysteine-rich CXC domain, and a C-terminal SET (Su(var)3-9, Enhancer of zeste, Trithorax) domain that contains the methyltransferase catalytic site (39–41). The EZH2 SET domain adopts the canonical methyltransferase fold, where conserved aromatic residues, including the catalytic Tyr726, contribute to formation of the methyl-lysine recognition channel adjacent to the S-adenosyl-L-methionine (SAM)-binding site. Structural studies further show that isolated EZH2 catalytic domains exhibit incomplete cofactor and substrate-binding configurations due to atypical positioning of the I-SET and post-SET regions, highlighting the requirement of PRC2 assembly for full catalytic activity (40, 42). Critically, EZH2 is enzymatically inert in isolation and acquires robust histone methyltransferase activity only after assembling with the non-catalytic core subunits SUZ12 and EED, which stabilize the SET domain and induce the conformational rearrangements required for full catalytic activation (43–45), RBBP4/7 (RbAp48/46) (46) and accessory factors, including AEBP2 (47), JARID2 (48), and PCLs (49), further regulate PRC2 recruitment, enzymatic activity, and chromatin-context sensitivity. Within the PCL family, MTF2 (PCL2) and PHF1 (PCL1) engage unmethylated CpG-containing DNA through an extended winged-helix module, providing a sequence-based mechanism for PRC2.1 recruitment (49), whereas EED binding to pre-existing H3K27me3 allosterically stimulates PRC2 catalysis and enables propagation of the repressive mark (50). Once deposited, H3K27me3 recruits PRC1, which monoubiquitinates histone H2A at lysine 119, to reinforce chromatin compaction and stable transcriptional silencing (51).

EZH2 dysregulation in cancer arises through both genetic and posttranslational mechanisms. Recurrent heterozygous gain-of-function mutations affecting the SET-domain residue tyrosine 641 (Y641F/N/S/H) are observed in approximately 22% of GCB-DLBCL and approximately 7% of follicular lymphomas (FL) in the original discovery cohort (52), with higher frequencies reported in subsequent follicular lymphoma cohorts, reaching approximately 27% in some series (53). Functionally, these mutations alter the catalytic properties of EZH2 by increasing its preference for dimethylated H3K27 substrates and cooperatively enhancing H3K27 trimethylation levels in the presence of wild-type EZH2 (54, 55). The less frequent A677G (56) and A687V (57) variants are also gain-of-function alterations that converge on H3K27 hypertrimethylation, but they do so through distinct changes in substrate preference. A677G exhibits approximately equal catalytic efficiency toward unmethylated, monomethylated, and dimethylated H3K27 (57), whereas A687V shows markedly increased activity toward monomethylated H3K27 with little gain toward the dimethylated substrate, and consequently increases H3K27me3 without the depletion of H3K27me2 seen with Y641 and A677 mutants (57). Catalytic activity is further fine-tuned by post-translational modifications: AKT-mediated phosphorylation at Ser21 redirects EZH2 toward non-histone substrates and converts it from a polycomb repressor into a transcriptional co-activator (58, 59), CDK1/CDK2-mediated phosphorylation at Thr350 supports H3K27me3 deposition (60), and AMPK-mediated phosphorylation at Thr311 disrupts EZH2–SUZ12 binding and attenuates PRC2 catalytic output (61).

Beyond its canonical PRC2-dependent silencing role, EZH2 exerts methylation- and PRC2-independent functions, behaving as a transcriptional co-activator through direct interactions with the androgen receptor (59), NF-κB subunits RelA/RelB (62), β-catenin (63), STAT3 (64), and c-MYC (65), and through direct methylation of non-histone substrates such as GATA4 (66) and RORα (67), with additional non-canonical PRC2-independent regulation of transcription factors such as PLZF (68).

### 2.2 EZH2 within the broader epigenetic network

EZH2 should therefore be considered within an interconnected epigenetic network rather than as an isolated chromatin-modifying enzyme, because its functional output can be shaped by paralogous PRC2 catalytic activity, DNA methylation, histone deacetylation, H3K27 demethylation, and ATP-dependent chromatin remodeling (45, 69–72). Within PRC2 itself, EZH1 can partially compensate for EZH2 in a context-dependent manner: genetic studies demonstrated proliferation-dependent EZH1/EZH2 redundancy, and in MHC-I-low cancer models, additional EZH1 deletion further increased MHC-I expression in EZH2-null cells (24, 45). These observations indicate that residual EZH1-containing PRC2 can preserve H3K27 methylation in selected biological contexts after EZH2 loss, providing a mechanistic rationale for distinguishing selective EZH2 inhibition from dual EZH1/EZH2 targeting rather than treating these interventions as equivalent (24, 45). EZH2/PRC2 can also intersect with DNA methylation: EZH2-containing Polycomb complexes have been shown to interact with DNA methyltransferases and to facilitate DNMT recruitment at selected repressed genes, linking H3K27 methylation to a second, potentially reinforcing layer of transcriptional silencing (69). The immune relevance of this interaction is illustrated in ovarian cancer, where EZH2-mediated H3K27me3 and DNMT1-mediated DNA methylation cooperatively repressed CXCL9 and CXCL10, thereby limiting effector T-cell trafficking and providing a mechanistic basis for combined disruption of histone- and DNA-methylation-dependent repression (21). Histone deacetylation constitutes a further reinforcing layer: EED-containing Polycomb complexes can associate with HDAC activity, indicating that loss of histone acetylation and gain of Polycomb-mediated H3K27 methylation can participate in coordinated transcriptional repression rather than functioning as completely independent chromatin processes (70). Tumor-immune studies provide more context-specific examples of this crosstalk: HDAC10 was required for EZH2 recruitment to the CXCL10 promoter in hepatic tumor cells, whereas combined EZH2 and HDAC inhibition further enhanced human leukocyte antigen class I (HLA-I) re-expression in Merkel cell carcinoma (73, 74). In contrast to the cooperative relationships with DNA methylation and histone deacetylation, PRC2 frequently opposes ATP-dependent SWI/SNF chromatin remodeling: whereas genetic *Ezh2* inactivation blocked tumour formation driven by *Snf5* loss (71). This antagonism can create genotype-specific dependencies: in ARID1A-mutated ovarian cancer models, pharmacological inhibition of EZH2 methyltransferase activity produced synthetic lethality, with PIK3IP1 identified as a shared ARID1A/EZH2-regulated target contributing to suppression of PI3K–AKT signaling (75). The relationship is nevertheless not uniform across all SWI/SNF alterations: in a subset of SMARCA4-mutant cancer models, sensitivity to EZH2 inhibition was associated with low SMARCA2 expression, and PRC2-mediated SMARCA2 repression contributed to the therapeutic dependency (76). At the same H3K27 regulatory node, the demethylase KDM6A functionally counteracts EZH2, and preclinical muscle-invasive bladder cancer models harboring KDM6A and/or SWI/SNF alterations showed sensitivity to EZH2 inhibition accompanied by increased IFN-γ- and NK-cell-associated signaling and tumor-cell differentiation (72). Collectively, these findings place EZH2 at a junction between partially redundant H3K27 methyltransferases, DNA methylation, histone deacetylation, H3K27 demethylation, and antagonistic SWI/SNF remodeling, indicating that the immunologic consequences of EZH2 targeting are likely to depend on the broader epigenetic state of the tumor rather than on EZH2 abundance alone (21, 24, 45, 69–71, 73–76). Accordingly, combinations of EZH2-directed therapy with DNMT or HDAC inhibition, as well as biomarker enrichment based on SWI/SNF or KDM6A alterations, should be regarded as mechanistically distinct strategies that require tumor-context-specific validation rather than as interchangeable approaches to achieving deeper epigenetic suppression (21, 71–76).

## 3 EZH2 as a prognostic and predictive biomarker in the immuno-oncology era

### 3.1 Correlative landscape: EZH2 and PD-L1 co-expression across tumor types

Across human tumor cohorts, the association between EZH2 and PD-L1 is heterogeneous and should currently be interpreted as correlative rather than evidence of a uniform regulatory relationship (77–79). In resected lung adenocarcinoma (n = 428), EZH2 and PD-L1 expression were positively associated, and their co-expression was linked to adverse clinicopathological features and shorter recurrence-free and overall survival (77). In uveal melanoma, computational analyses linked EZH2 expression to immune-checkpoint transcripts, immune-cell infiltration, and Tumor Immune Dysfunction and Exclusion (TIDE)-predicted immunotherapy resistance, while an independent cohort supported an association between EZH2 and PD-L1 expression (78). In hepatocellular carcinoma, ncRNA-associated EZH2 upregulation was correlated with the composition of the tumor immune infiltrate, although these data did not establish a causal mechanism linking EZH2 to checkpoint regulation (80).

Conversely, in endometrial carcinoma, PD-L1 and EZH2 expression are largely mutually exclusive, and the PD-L1^-^/EZH2^+^ phenotype is associated with the least favorable survival profile among the evaluated expression groups (79). Collectively, these studies indicate that EZH2–PD-L1 relationships are tumor-context dependent and are not sufficiently consistent or prospectively validated to support EZH2–PD-L1 co-expression as a decision-ready biomarker. The mechanistic basis of EZH2-dependent PD-L1 regulation is considered separately in Section 4.2.

### 3.2 Clinical-correlative relationships between EZH2 and CD8^+^ T-cell infiltration

Human correlative studies do not support a uniformly inverse relationship between tumor EZH2 expression and CD8^+^ T-cell abundance, indicating that EZH2 expression alone cannot be interpreted as a surrogate marker of an immune-excluded tumor state (81, 82). In oral leukoplakia, epithelial EZH2 expression correlated positively with CD3^+^ and CD8^+^ T-cell infiltration and was associated with an increased risk of subsequent malignant transformation (81). Similarly, an analysis of more than 1,600 renal cell carcinomas demonstrated a positive association between EZH2 expression and CD8^+^ T-cell density, while high EZH2 expression and CD8^+^ density were each associated with unfavorable clinicopathological features and outcomes (82). Transcriptomic analyses in lung adenocarcinoma have associated EZH2 expression with cell cycle-related programs and variations in immune cell infiltration; however, these data are primarily correlative and do not establish EZH2 as a causal regulator of the observed immune composition (83). In skull base chordoma, higher EZH2 expression is associated with tumor stemness, immune checkpoint-related features, and increased CD163^+^ macrophage infiltration, further illustrating that EZH2 abundance can coexist with complex rather than uniformly excluded immune microenvironments (84). Thus, current biomarker studies indicate that the relationship between EZH2 expression and CD8^+^ T-cell infiltration is tumor-context-dependent and cannot distinguish functional immune exclusion from the presence of quantitatively abundant but functionally uncharacterized T-cells. The experimentally supported mechanisms regulating CD8^+^ T-cell recruitment and exclusion are discussed separately in Section 4.3.

### 3.3 Toward biomarker-guided patient selection: an actionable multi-domain framework

A central lesson emerging from contemporary biomarker discovery is that EZH2 protein or transcript abundance is a poor proxy for its functional impact on tumor immunity, a realization that is reshaping the stratification of patients for epigenetic–immune combination therapy. In olfactory neuroblastoma, EZH2 transcripts are nearly threefold higher than in epithelioid sarcoma, the tumour type in which tazemetostat held an accelerated-approval indication until its withdrawal in 2026 (Section 7); however, the inferred methyltransferase activity, captured by the EZH2 Repression Signature (ERS), is indistinguishable between the two tumors. EZH2 expression and ERS are essentially uncorrelated (r ≈ 0.1), and EZH2-high tumors show no differential immune infiltrate, T cell-inflamed (TCI) score, or checkpoint gene expression (85). In stark contrast, ERS-defined low-activity tumors, regardless of expression, are enriched for β2M, PD-L1, CTLA-4, PD-1, VISTA, and T cell-inflamed phenotypes, suggesting that activity-based metrics may align more closely than expression-based metrics with the immune context relevant to therapy; this association is retrospective, derived from a single tumour type, and has not been prospectively tested (85, 86). This conceptual shift is further supported by a computational machine-learning multi-omic analysis of hepatocellular carcinoma, in which EZH2 was incorporated into a CEP55–DLGAP5–EZH2 regulated cell-death signature associated with predicted immunotherapy resistance (87). Collectively, these observations argue against the use of EZH2 transcript or protein abundance as a stand-alone selection biomarker and instead support a multidimensional framework that integrates functional EZH2/PRC2 activity with the immune phenotype of the tumor (74, 85). Operationally, we propose a research-use baseline panel comprising four domains: (i) functional EZH2/PRC2 activity, assessed by an RNA-based EZH2 repression/activity signature, such as ERS, together with tumor H3K27me3; (ii) antigen-presentation competence, assessed by HLA class I and β2-microglobulin (β2M), with CIITA/MHC-II included where interferon-γ-inducible MHC-II is biologically relevant; (iii) interferon and chemokine competence, assessed by type I/II interferon-response programs and CXCL9/CXCL10, with tumor-context-specific interrogation of STING or MAVS where the proposed mechanism involves viral mimicry; and (iv) immune contexture, assessed by CD8^+^ T-cell abundance together with regulatory T-cell and suppressive myeloid features using multiplex or spatial immune profiling (21, 24, 29, 74, 85, 88–94). PD-L1 should be recorded as a contextual variable rather than used as a stand-alone gate for this panel because the direction of EZH2-dependent PD-L1 regulation differs across tumor contexts and can involve distinct transcriptional or post-translational mechanisms (95–98). As a prospective trial-enrichment hypothesis, tumors showing evidence of high functional EZH2/PRC2 activity together with potentially reversible immune repression, such as reduced antigen presentation or attenuated interferon/TH1-chemokine programs, would constitute the most mechanistically informative group in which to test EZH2 inhibitor–checkpoint blockade combinations, whereas the presence of a dominant suppressive myeloid phenotype should be flagged as a potential competing liability rather than assumed to predict benefit (21, 24, 29, 30, 88–90, 93, 99). A paired early on-treatment biopsy could then be used to confirm pharmacodynamic target engagement through reduced H3K27me3 and to determine whether this engagement is accompanied by a tumor-context-appropriate immune change, such as increased HLA-I/β2M, increased CIITA/MHC-II inducibility, or restoration of CXCL9/CXCL10 expression (21, 74, 88, 100). In the studies reviewed here, no prospective trial has validated cutoffs, relative weights, or a composite score for these variables; accordingly, this framework should be regarded as a trial-enrichment and biomarker-development schema rather than a clinical decision rule, and prospective studies should test whether baseline and on-treatment composite readouts predict incremental benefit from EZH2 inhibition beyond checkpoint blockade alone (101–103).

## 4 Mechanistic layers of EZH2-driven tumor-intrinsic immune evasion

### 4.1 Silencing of MHC class I and II antigen presentation machinery

At the mechanistic level, Burr et al. defined a PRC2-dependent (complex-level) program, rather than a PRC2-independent function of EZH2, as the principal epigenetic mechanism coordinating the repression of the MHC class I antigen-processing pathway (MHC-I APP) (24). A genome-wide CRISPR/Cas9 screen performed in MHC-I-low K-562 cells recovered core PRC2 subunits (EED, SUZ12, and EZH2) together with the PRC2.1 accessory component MTF2 as negative regulators of MHC-I, placing the causative unit at the level of the assembled complex rather than at EZH2 alone (24). In MHC-I-low cancers, MHC-I APP gene promoters carried bivalent activating H3K4me3 and repressive H3K27me3 modifications, a configuration that suppressed basal MHC-I expression and additionally restricted cytokine-inducible upregulation (24). Importantly, EZH2 catalytic activity contributes to this canonical PRC2-dependent mechanism: pharmacological inhibition of EED or EZH1/2 relieved MHC-I repression, whereas catalytically inactive EZH2 could not support PRC2-mediated repression of MHC-I (24). Accordingly, study (24) supports an EZH2 catalytic contribution to PRC2-dependent MHC-I silencing but does not establish a distinct PRC2-independent EZH2 mechanism at the MHC-I APP. Mechanistically, these findings support a coordinated model in which PRC2 constitutes the functional repressive complex, whereas EZH2 provides the H3K27 methyltransferase activity required to establish and maintain the repressive chromatin state (24). Within this complex, EED and SUZ12 are required for efficient EZH2 catalytic activity, and EZH2-dependent H3K27 methylation contributes to the H3K4me3/H3K27me3 bivalent state at MHC-I antigen-processing pathway loci, thereby restricting both basal transcription and cytokine-inducible expression (24, 43, 44). The complex-level nature of this mechanism is further supported by the observation that EED loss produced greater MHC-I derepression than EZH2 loss and that additional EZH1 deletion further increased MHC-I expression in EZH2-null cells, indicating partial compensation by EZH1-containing PRC2 complexes (24). Importantly, mutation of the catalytic EZH2 SET-domain residue F667 abolished the ability of EZH2 to restore MHC-I repression, establishing a requirement for EZH2 methyltransferase activity within this PRC2-dependent pathway (24).

In contrast to the complex-level mechanism established by Burr et al. (24), subsequent studies have provided EZH2-focused functional evidence through genetic manipulation of EZH2, modulation of EZH2 protein stability or reconstitution, and pharmacological inhibition of its catalytic activity in defined tumor contexts (74, 100, 104, 105). One such EZH2-dependent regulatory axis involves USP22-mediated deubiquitination and stabilization of EZH2, thereby increasing EZH2 protein abundance and promoting H3K27me3-associated repression of MHC-I genes (104). Genetic USP22 ablation increased MHC-I expression and enhanced CD8^+^ T-cell activation and cytotoxicity, whereas reconstitution with wild-type EZH2, but not the catalytically inactive F667I mutant or a SET-domain-deficient EZH2 construct, reversed these effects (104). These rescue experiments provide EZH2-specific functional evidence that its methyltransferase activity is required for MHC-I repression in the USP22-dependent setting, while remaining consistent with EZH2 functioning through its canonical chromatin-modifying machinery (104). In oral potentially malignant disorders, complementary EZH2 knockdown and pharmacological inhibition experiments linked EZH2 activity to H3K27me3-mediated repression of MHC-I antigen-processing machinery, including HLA-A/B/C, *B2M*, and TAP1 (105). EZH2 inhibition reduced H3K27me3 and restored expression of MHC-I antigen-processing genes, supporting an EZH2-dependent epigenetic mechanism during early oral carcinogenesis (105). In human papillomavirus (HPV)-negative head and neck squamous cell carcinoma (HNSCC), both pharmacological EZH2 inhibition and CRISPR/Cas9-mediated EZH2 deficiency increased MHC-I expression and enhanced antigen-specific CD8^+^ T-cell proliferation, IFN-γ production, and tumor-cell cytotoxicity (100). Mechanistically, pharmacological EZH2 inhibition reduced H3K27me3 enrichment at the *B2M* promoter, linking EZH2 catalytic activity to epigenetic control of MHC-I antigen presentation in this tumor context (100). Because these experiments demonstrate genetic and pharmacological dependence on EZH2 but do not by themselves establish direct physical occupancy of EZH2 at the *B2M* promoter, this mechanism is most precisely described as EZH2-dependent rather than as direct EZH2 promoter binding (100).

Notably, Merkel cell carcinoma provides an important mechanistic refinement: HLA-I loss is correlated with H3K27me3 abundance but not with EZH2 protein expression, indicating that EZH2 abundance alone may not accurately reflect the functional state of PRC2-mediated repression (74). Complementary preclinical melanoma data showed that prolonged metabolic stress reduced EZH2 abundance and H3K27me3 at MHC-I antigen-presentation loci, increased MHC-I expression, and enhanced CD8^+^ T-cell-mediated killing, further linking metabolic adaptation to the epigenetic regulation of tumor antigenicity (106). Treatment with tazemetostat and DZNep reduced H3K27me3 and restored surface HLA-I expression, with further enhancement observed when EZH2 and histone deacetylase inhibition were combined (74). However, several important limitations of this model should be considered. In this tumor context, H3K27me3 abundance, reflecting PRC2 catalytic output, tracked HLA-I loss more closely than EZH2 protein expression, and restoration of antigen-processing machinery components may not by itself be sufficient for effective T-cell recognition (24, 74, 100).

Collectively, the available evidence supports two mechanistically related but non-equivalent levels of regulation: a canonical PRC2-dependent program in which EZH2 provides H3K27 methyltransferase activity and tumor-context-specific observations obtained through direct genetic or pharmacological perturbation of EZH2 (**Figure 1**). These categories should not be treated as mechanistically interchangeable, and the findings of Burr et al. (24) should be interpreted as demonstrating PRC2-dependent MHC-I repression with a required catalytic contribution from EZH2, rather than a PRC2-independent EZH2 function.

**Figure 1:**
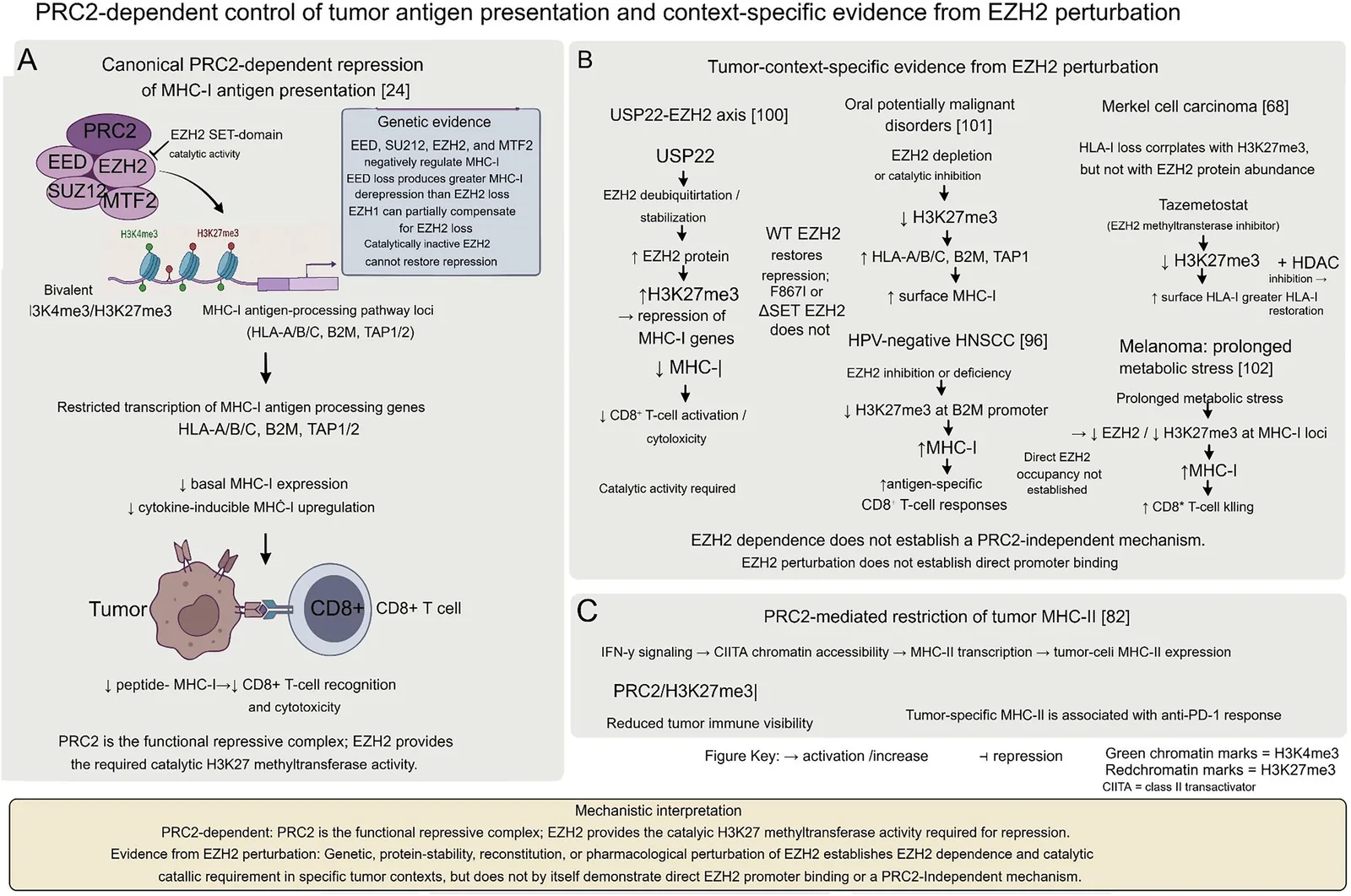
PRC2-dependent and EZH2-directed regulation of MHC class I and II antigen presentation. **(A)** In MHC-I-low tumors, PRC2 components EED, SUZ12, EZH2, and MTF2 maintain H3K27me3-containing bivalent chromatin at MHC-I antigen-processing loci, thereby restricting basal and cytokine-inducible MHC-I expression. EZH2 provides the catalytic H3K27 methyltransferase activity within this PRC2-dependent mechanism; however, the evidence does not establish a separate PRC2-independent EZH2 pathway (24). **(B)** Tumor-context-specific EZH2 perturbation alters MHC-I antigen presentation through changes in EZH2 stability or catalytic activity. USP22-mediated EZH2 stabilization promotes MHC-I repression, whereas EZH2 depletion or inhibition restores antigen-presentation machinery in oral premalignant lesions and HNSCC; H3K27me3 activity also tracks HLA-I regulation in Merkel cell carcinoma and metabolically stressed melanoma (74, 100, 104–106). **(C)** PRC2-mediated H3K27me3 restricts IFN-γ-inducible *CIITA* accessibility and tumor-cell MHC-II expression (88). Arrows indicate activation/increase and blunt-ended lines indicate repression.

### 4.2 The EZH2–PD-L1 axis: a bidirectional, tumor-context–dependent regulator

The following subsection is restricted to mechanistic evidence defining how EZH2 regulates PD-L1; the heterogeneous human correlative relationships between EZH2 and PD-L1 are summarized separately in Section 3.1. At the transcriptional level, EZH2 can function as a direct *repressor* of immune checkpoints. In hepatocellular carcinoma (HCC), EZH2 deposits H3K27me3 at the promoters of both *CD274* and *IRF1*, the master IFN-γ-responsive transactivator of PD-L1, suppressing PD-L1 in an IFN-γ-dependent manner without altering STAT1 activation, with an inverse EZH2–PD-L1 correlation confirmed in immune-activated HCC tissues (95). In non-small cell lung cancer (NSCLC), EZH2 regulation of PD-L1 proceeds in the opposite direction and is dependent on hypoxia-inducible factor-1α (HIF-1α) (96). EZH2 knockdown reduced HIF-1α expression together with PD-L1 mRNA and protein, whereas hypoxic exposure increased HIF-1α and PD-L1; importantly, EZH2 knockdown attenuated PD-L1 expression even under hypoxic conditions (96). These findings support an EZH2→HIF-1α→PD-L1 regulatory relationship in this model, while hypoxia represents a parallel inducer of HIF-1α rather than an experimentally demonstrated upstream activator of EZH2 (96). A distinct lineage-associated circuit has been reported in triple-negative breast cancer (TNBC), where FEZF2 overexpression reduced EZH2 and PD-L1 expression in MDA-MB-231 and BT-549 cells; EZH2 overexpression restored PD-L1 expression, whereas PD-L1 overexpression attenuated the growth-inhibitory effect of FEZF2 overexpression (107). In xenograft models, FEZF2 overexpression was also associated with increased CD8^+^ tumor-infiltrating lymphocytes (107). These experiments support an FEZF2–EZH2–PD-L1 regulatory relationship but do not directly establish the consequences of genetic FEZF2 loss.

Beyond transcriptional regulation, a recent study in TNBC, including the brain-metastatic 231Br model, linked EZH2 to the abundance of N-linked glycosylated PD-L1 (97). N-linked glycosylation is functionally relevant because prior mechanistic work demonstrated that glycosylated PD-L1 is more stable than its non-glycosylated counterpart; glycosylation antagonizes GSK3β binding to PD-L1, thereby limiting GSK3β-dependent phosphorylation and subsequent β-TrCP-mediated ubiquitination and proteasomal degradation, ultimately preserving PD-L1-mediated immunosuppressive activity and restraining T-cell activity (108). In the TNBC brain-metastasis study, EZH2 knockdown reduced both overall PD-L1 abundance and the N-glycosylated PD-L1 species, while tunicamycin, an inhibitor of N-linked glycosylation, reduced glycosylated PD-L1 without altering EZH2 levels (97). Co-immunoprecipitation further showed that EZH2 and PD-L1 were present in the same protein complex in MDA-MB-231 and brain-metastatic 231Br cells; however, this assay demonstrates protein association and does not by itself establish direct molecular binding or the biochemical mechanism by which EZH2 influences PD-L1 glycosylation (97). Capsanthin treatment decreased EZH2, total PD-L1, and N-glycosylated PD-L1 and was accompanied by reduced migration and epithelial–mesenchymal transition-associated features in the experimental TNBC models (97). These findings provide a preclinical rationale for investigating whether modulation of the EZH2–PD-L1/glycosylation relationship can alter immune checkpoint function; however, this study (97) did not directly measure PD-L1 protein half-life, T-cell-mediated cytotoxicity, or therapeutic synergy between capsanthin and PD-1/PD-L1 blockade. A distinct post-translational mechanism has been reported in colorectal cancer models, in which EZH2 inhibition increased USP22 expression and promoted USP22-mediated deubiquitination and stabilization of PD-L1; combined EZH2 inhibition and PD-1 blockade subsequently produced greater antitumor activity than either intervention alone in the experimental models (109). Notably, USP22 appears in these two settings with opposite directionality: in the MHC-I context it acts upstream to stabilize EZH2 and reinforce antigen-presentation silencing (104), whereas in colorectal cancer models it is induced downstream of EZH2 inhibition and stabilizes PD-L1 (109). Because these observations derive from different tumor types and perturbation designs, they should not be assumed to describe a single reciprocal circuit; testing EZH2 and USP22 perturbation within the same model would be required to determine whether a feedback relationship exists and whether USP22 induction represents an adaptive response limiting the benefit of EZH2 inhibition.

In Burkitt lymphoma models with low baseline PD-L1 expression, treatment with the EZH2 degrader MS1943 reduced EZH2 protein abundance and produced a modest increase in PD-L1 expression, with a more evident PD-L1 increase observed during co-culture with activated CD8^+^ T cells (98). EZH2 degradation is also associated with increased NF-κB and IRF1 levels, which are implicated in the accompanying increase in PD-L1 expression (98). Combining MS1943 with anti-PD-1 produced greater antitumor activity than either treatment alone in CD8^+^ T-cell co-culture and a human PBMC-reconstituted lymphoma xenograft model (98). However, because the study did not establish that PD-L1 induction itself was necessary for the enhanced combination effect, these data support an association between EZH2 degradation, PD-L1 induction, and sensitivity to the combined anti-PD-1 treatment rather than a definitive causal sequence (98).

Taken together, PD-L1 regulation by EZH2 is context-dependent and can reflect distinct transcriptional and post-translational mechanisms, with experimentally supported determinants including the IFN-γ-responsive state, HIF-1α signaling, lineage-specific transcriptional programs, and PD-L1 post-translational modification (**Figure 2**). Most evidence remains *in vitro* and single-tumor; clinically, these findings argue for biomarker-guided stratification, not universal combination, before pairing EZH2 inhibitors with PD-1/PD-L1 blockade therapy.

**Figure 2:**
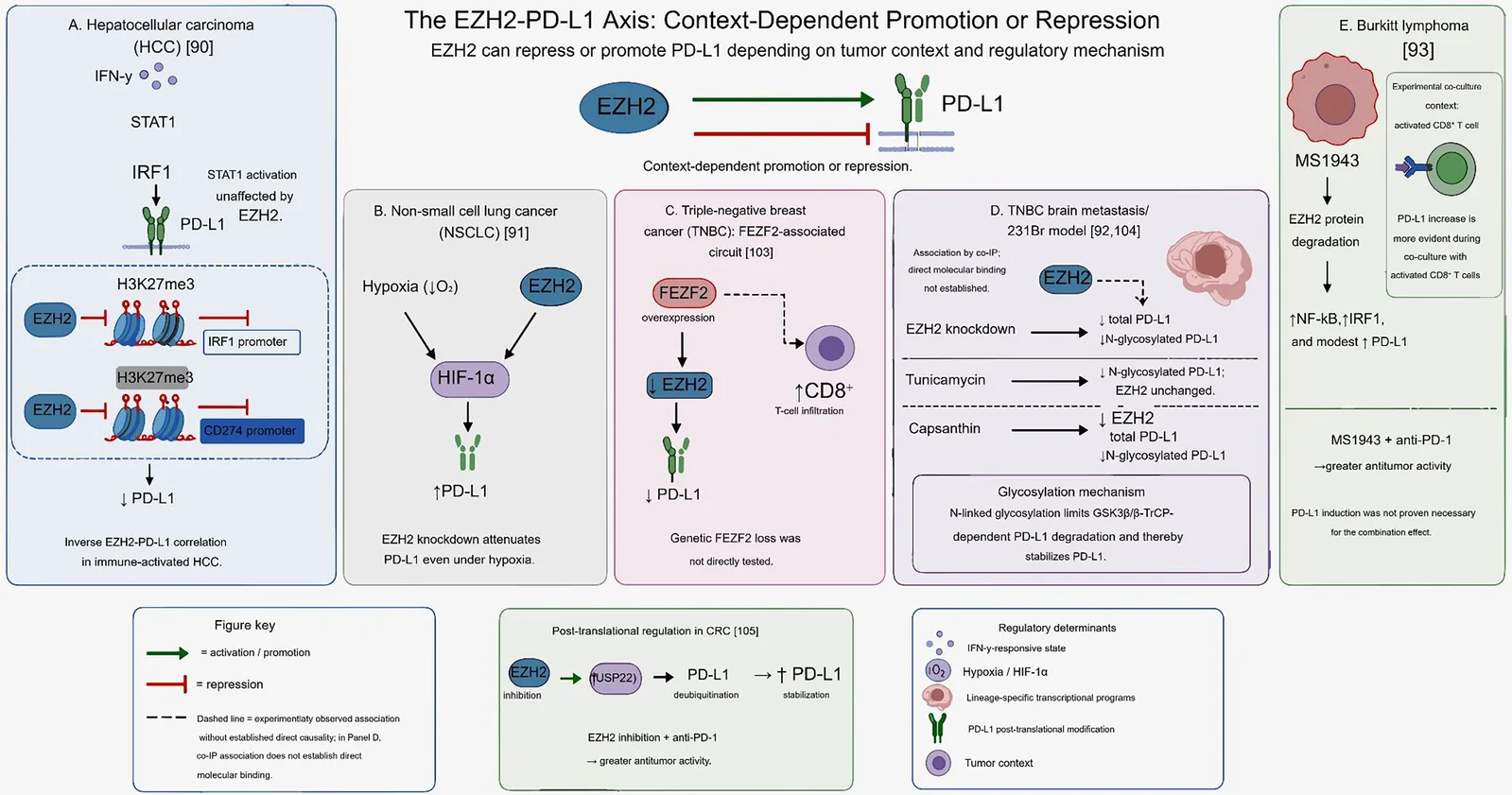
Context-dependent bidirectional regulation of the EZH2–PD-L1 axis. **(A)** In hepatocellular carcinoma (HCC), EZH2-mediated H3K27me3 at the *IRF1* and *CD274* promoters represses IFN-γ-responsive PD-L1 expression without altering STAT1 activation (95). **(B)** In non-small cell lung cancer (NSCLC), EZH2 promotes PD-L1 expression through HIF-1α, whereas hypoxia independently induces HIF-1α (96). **(C)** In triple-negative breast cancer (TNBC), FEZF2 overexpression reduces EZH2 and PD-L1 levels and is associated with increased CD8^+^ T-cell infiltration; genetic FEZF2 loss was not directly tested (107). **(D)** In TNBC/231Br models, EZH2 is associated with total and N-glycosylated PD-L1. Capsanthin and tunicamycin reduce PD-L1 glycosylation, whereas N-linked glycosylation stabilizes PD-L1 by limiting GSK3β/β-TrCP-dependent degradation (97, 108). **(E)** In Burkitt lymphoma, MS1943-mediated EZH2 degradation is associated with increased NF-κB, IRF1, and PD-L1 levels, and enhances the antitumor activity of anti-PD-1 (98). In colorectal cancer, EZH2 inhibition promotes USP22-mediated PD-L1 deubiquitination and stabilization (109). Overall, EZH2-dependent PD-L1 regulation varies according to the tumor context and regulatory mechanisms.

### 4.3 Chemokine silencing and CD8^+^ T-cell exclusion: “turning off the recruiting signal”

The following subsection focuses on experimentally supported mechanisms governing CD8^+^ T-cell recruitment and exclusion. The correlative relationships between EZH2 expression and CD8^+^ T-cell abundance in human tumors are summarized in Section 3.2. The trafficking of effector CD8^+^ T cells into tumors is governed by TH1-type chemokine gradients, whose silencing through coordinate chromatin repression constitutes a foundational and therapeutically reversible axis for immune evasion. A landmark ovarian cancer study established that EZH2-mediated H3K27me3 and DNMT1-mediated DNA methylation cooperatively repress *CXCL9* and *CXCL10*, inversely correlating with tumor-infiltrating CD8^+^ cells, and that epigenetic reprogramming restores effector cell trafficking while synergizing with anti-PD-L1 blockade and adoptive T cell transfer (21). This paradigm has been supported and further refined in several epithelial malignancies. In esophageal squamous cell carcinoma, tumor-intrinsic EZH2 produces a prototypical CD8^+^ T-cell desert through a dual circuit: direct disruption of NF-κB-driven *CXCL9* transactivation, compounded by elevated VEGFC secretion, which impairs dendritic cell maturation. This suggests that chemokine silencing may be reinforced rather than acting in isolation by the parallel suppression of antigen-presenting accessory compartments (110). In lung adenocarcinoma, EZH2 inhibition coordinately de-represses *CCL5* and MHC class I, restoring both CD8^+^ chemotaxis and antigen recognition without altering PD-L1, supporting a model in which EZH2 constrains both lymphocyte recruitment and antigen recognition, processes that may need to be restored in parallel for maximal therapeutic benefit (26).

Upstream of direct chromatin deposition, EZH2 activity is amplified by stabilizing and scaffolding partners that become actionable nodes. In colon cancer, wild-type NCOA3 acetylates H3K27 at the *HSP90α* promoter, and the resulting HSP90α pool stabilizes EZH2, promoting *CXCL9* repression and anti-PD-L1 resistance. NCOA3 loss-of-function mutations rescue CD8^+^ T-cell infiltration and ICB response (111). In gastric cancer, long non-coding RNA LINC00152 physically recruits EZH2 to *CXCL9/CXCL10* promoters, engaging the CXCR3 trafficking axis, with CD8^+^ depletion nullifying the antitumor effect of LINC00152 silencing, supporting a causal role for this lncRNA–EZH2 axis in regulating T cell infiltration (112).

Importantly, EZH2-dependent chemokine regulation may not only limit lymphocyte recruitment but also contribute to dysfunctional T-cell states once immune cells are present, in some contexts. In myelodysplastic syndrome (MDS)-to-AML transformation, EZH2/H3K27me3-mediated CXCL10 loss promotes CD8^+^ T-cell exhaustion, distinguishing a *pseudo-inflamed* dysfunctional state from true immune exclusion (113). Across these models, EZH2 contributes to TH1-chemokine repression through mechanistically distinct entry points, direct chromatin deposition, lncRNA scaffolding, and cofactor-mediated protein stabilization, that converge on a recruitment-deficient phenotype. None of the cited studies assessed whether this repression confers a selective advantage during tumor evolution; this should therefore be treated as a hypothesis rather than an established property (21, 26, 110–113). Current evidence remains largely preclinical and lacks spatial single-cell validation; translationally, EZH2 inhibitor–ICB combinations will require pre-treatment stratification by whether tumors are recruitment-deficient or post-recruitment dysfunctional.

### 4.4 Reawakening viral mimicry: the dsRNA–STING–interferon axis

The viral mimicry paradigm reframes EZH2 not merely as a silencer of immune effector genes but as the gatekeeper of an endogenous innate immune trigger, coupling chromatin repression of retroelements to the entire type I IFN program. At baseline, PRC2/EZH2 deposits H3K27me3 across endogenous retroviruses (ERVs), repetitive elements, and the IFN axis, preventing the generation of cytosolic double-stranded RNA (dsRNA) and maintaining a transcriptional state that is incompatible with innate sensing. In several preclinical models, pharmacological EZH2 inhibition reverses this state through a broadly similar sequence, endogenous retrovirus de-repression, dsRNA accumulation, pattern-recognition receptor engagement, interferon signaling, and downstream antigen-presentation and chemokine reactivation, accompanied by increased T-cell infiltration and enhanced sensitivity to checkpoint blockade in the systems examined (89, 90, 92). Whether this constitutes a generalizable conversion of immune-cold human tumors to a checkpoint-responsive state has not been establish.

In non-small cell lung cancer, EZH2 inhibition has been reported to increase dsRNA accumulation with downstream signaling dependent on TLR3 and MDA5, activating an interferon response that coordinately upregulated antigen-presenting machinery and PD-L1, increased CD8^+^ T-cell infiltration, and enhanced the activity of anti-PD-1 in the models examined (89). In prostate cancer, an analogous circuit converges on STING: EZH2 inhibition activates a dsRNA–STING–interferon-stimulated gene (ISG) axis that is genetically STING-dependent and reverses PD-1 resistance with concurrent M1 TAM polarization (90), illustrating that “viral mimicry” is a convergent innate-immune state accessed through tumor-context–specific sensors rather than through a single pathway. Beyond retroelement de-repression, PRC2 directly silences the IFN program itself: in ERα^+^ luminal breast cancer, H3K27me3 blankets type I IFN ligand promoters, and EZH2 inhibition establish an autocrine IFN–STAT2, not STAT1, axis that defines immune responsiveness (91). Extending these observations to chromatin architecture, a 2026 preprint (not yet peer reviewed) reported that dual EZH1/EZH2 inhibition in small cell lung cancer models reorganized 3D genome structure at the level of compartments, topologically associating domains, and enhancer–promoter loops, accompanied by reactivation of MHC class I and a neuroendocrine-to-non-neuroendocrine lineage shift; these findings require peer-reviewed confirmation (114). PTEN-deficient glioblastoma exposes pathway dependency: ERV–MAVS–IFN signaling is so deeply suppressed that DNMT inhibition alone fails, yet EZH2 inhibition combined with 5-azacytidine synergistically restores viral mimicry (92). Notably, STING itself is silenced by H3K27me3 in melanoma; EZH2 inhibition induces a switch from H3K27me3 to H3K27ac, thereby re-expressing STING and synergizing with STING agonists (93).

Collectively, these studies identify tumor-intrinsic EZH2/PRC2 as one regulator of an interferon-gated innate-sensing program in the models examined. Because none of these studies tested whether this program accounts for checkpoint-blockade resistance in patients, EZH2-dependent repression of innate sensing should be regarded as a candidate contributor rather than an established proximate driver of ICB resistance (89–93). However, tumor-type reliance on MAVS versus STING and intact IFN machinery determines the response; biomarker-guided stratification must precede combinations of EZH2 inhibition with ICB, DNMT inhibition, or STING agonists.

### 4.5 Emerging non-canonical layers — ncRNAs, circular EZH2, RNA editing, and senescence

The canonical framing of EZH2 as an H3K27me3 writer captures only the most visible tier of its influence on tumor immunity; emerging evidence positions it as a multi-modal immune regulator whose activities span non-coding RNA guidance, non-canonical protein isoforms, RNA-level editing, and secretome reprogramming, layers that converge on antigen presentation, innate sensing, NK-ligand display, and chemokine-driven recruitment.

Specificity in immune-gene silencing is frequently conferred by lncRNA scaffolding rather than by that of EZH2. A pan-cancer analysis of 9,626 tumors identified lincRNA EPIC1 as a direct EZH2 interactor that targets methyltransferase to the *IFNGR1*, *TAP1/2*, *ERAP1/2*, and MHC-I loci, with genetic or pharmacological EZH2 inhibition abolishing EPIC1’s immunosuppressive effect and restoring IFN-γ responsiveness (115). This establishes a paradigm in which EZH2’s immune impact is defined as much by its RNA-guided address as by its enzymatic output. Non-canonical protein isoforms extend this logic further: in glioblastoma, the circular EZH2 transcript is translated into a 92-amino-acid protein (EZH2-92aa) that directly binds *MICA/B* promoters to repress their transcription while concurrently stabilizing full-length EZH2 to suppress *ULBPs* — a dual mechanism that collapses the NKG2D ligand repertoire, mediates NK-resistance of glioblastoma stem cells, and synergizes with anti-PD-1 (116).

At the RNA level, EZH2 physically interacts with ADAR1 and competes with ILF2 to reshape A-to-I editing substrate selectivity and stabilize oncogenic transcripts via a TRN1–ADAR1p110 axis that is independent of methyltransferase activity (117). This explains why ADAR1 depletion sensitizes cancer cells to EZH2 degraders but not to catalytic inhibitors. At the secretome level, EZH2 represses proinflammatory senescence-associated secretory phenotype (SASP) genes (CCL2, CXCL9/10) following therapy-induced senescence in pancreatic cancer cells. EZH2 blockade converts senescent cells into immune-recruiting hubs that engage NK and T-cells (118). A conference-reported analysis has additionally proposed a reciprocal EZH2–RKIP axis as a further node linking EZH2 to immune-evasion signaling; this proposal has not yet been reported in a peer-reviewed format and requires independent validation (119).

These layers remain heterogeneous, tumour-specific, and incompletely unified, and the relative contribution of each is undefined. Collectively, however, they identify EZH2 functions that would not be engaged by catalytic inhibition alone, and provide a rationale for evaluating next-generation strategies that target EZH2 beyond its methyltransferase activity, including selective degraders such as PROTAC- or hydrophobic-tagging-based agents, circRNA-directed approaches, and peptide-mediated disruption of RNA–protein interfaces. Whether any of these approaches overcomes clinical resistance to catalytic EZH2 inhibitors has not been tested. Together, these studies illustrate the mechanistic diversity of tumor-intrinsic EZH2/PRC2-associated immune regulation, as summarized in **Table 1**.

**Table 1:** Mechanistic evidence for PRC2- and EZH2-associated tumor-intrinsic immune evasion.

Mechanistic layer	Cancer type/model	Key finding	Therapeutic outcome	Ref.
Silencing of MHC class I and II antigen presentation machinery	MHC-I-low cancer models; genome-wide CRISPR/Cas9 screen	Genome-wide CRISPR/Cas9 screening identified multiple PRC2 core/accessory components, including EED, SUZ12, EZH2, and MTF2, as negative regulators of the MHC-I antigen-presentation pathway. These findings support a PRC2-dependent, complex-level mechanism in which EZH2 provides the required H3K27 methyltransferase activity; however, they do not establish a distinct PRC2-independent EZH2 mechanism at these loci.	Genetic or pharmacological disruption of PRC2 activity restores MHC-I expression and increases susceptibility to T-cell-mediated immune recognition in MHC-I-low cancer models.	(24)
	Human lung cancer specimens (correlative analysis); syngeneic preclinical models	USP22 functioned as a deubiquitinase that stabilized EZH2 and silenced MHC-I; genetic and pharmacologic USP22 inhibition increased MHC-I and CD8^+^ T cell killing, abrogated by Ezh2 reconstitution.	Increased immunogenicity and overcame anti-PD-1 resistance; USP22 associated with ICB resistance in lung cancer patients.	(104)
	Oral leukoplakia/OPMDs → HNSCC; topical PPT@GSK126 hydrogel	EZH2 was negatively correlated with MHC-I and CD8^+^GZMB^+^ subsets in OPMDs; EZH2 triggered H3K27me3 on HLA-A/B/C, *B2M*, and TAP1 promoters.	opical PPT@GSK126 reduced malignant progression of oral premalignant lesions in mice and enhanced the effect of topical anti-PD-1.	(105)
	HPV-negative HNSCC cell lines; mouse anti-PD-1-resistant model	EZH2 inhibition (GSK126, EPZ6438) reduced H3K27me3 at the β-2-microglobulin promoter and upregulated MHC-I expression, increased antigen-specific CD8^+^ proliferation, IFN-γ production, and cytotoxicity.	Combination therapy with anti-PD-1 suppressed tumor growth in a resistant model.	(100)
	Merkel cell carcinoma TMA (n=118) + WaGa, PeTa, MKL1 lines	HLA-I loss was associated with high H3K27me3 but not EZH2 expression, and tazemetostat and DZNeP reduced H3K27me3 and restored surface HLA-I.	Combined EZH2 and HDAC inhibition further increased HLA-I re-expression.	(74)
	Human melanoma biopsies from anti-PD-1-treated patients (clinical-correlative/translational analysis); preclinical mechanistic experiments	PRC2/EZH2 and H3K27 hypermethylation suppressed tsMHC-II expression, while EZH2 inhibition improved chromatin accessibility at CIITA and MHC-II inducibility.	PRC2-related gene expression is inversely associated with anti-PD-1 response.	(88)
The EZH2–PD-L1 axis: a bidirectional, tumor-context–dependent regulator	Hepatocellular carcinoma cell lines and patient samples	EZH2 suppressed PD-L1 in an IFN-γ-dependent manner by depositing H3K27me3 on CD274 and IRF1 promoters; inverse EZH2–PD-L1 correlation in immune-activated HCC	In this retrospective cohort, combined EZH2/PD-L1 status was associated with OS and RFS on multivariable analysis; not prospectively validated.	(95)
	Burkitt’s, B-cell, CTCL, Hodgkin’s lymphoma lines; hu-PBMC-CDX	The hydrophobic-tagging-based selective EZH2 degrader MS1943 showed stronger anti-proliferative effects than catalytic EZH2 inhibition in lymphoma models, inducing EZH2 depletion, apoptosis, and modest PD-L1 upregulation that enhanced antitumor activity with PD-1 blockade.	Synergistic antitumor effect of EZH2 degrader + anti-PD-1 in humanized model	(98)
	NSCLC cell lines and *in vivo* xenografts	EZH2 was positively correlated with PD-L1. shRNA knockdown suppressed PD-L1 mRNA and protein expression via HIF-1α dependence.	Delayed lung cancer progression and enhanced antitumor immune responses	(96)
	TNBC and brain-metastatic TNBC cells + MSCs (3A6)	Capsanthin reduced EZH2 and N-linked glycosylated PD-L1; co-immunoprecipitation detected EZH2 and PD-L1 within the same protein complex; direct molecular binding was not established.	Inhibited migration and EMT markers in brain-metastatic TNBC	(97)
	TNBC (MDA-MB-231, BT-549) xenografts	FEZF2 overexpression decreased EZH2 and PD-L1; EZH2 overexpression restored PD-L1 expression, whereas PD-L1 overexpression attenuated the growth-inhibitory effect of FEZF2 overexpression. Genetic FEZF2 loss was not directly tested.	FEZF2 overexpression reduced tumor weight and volume and was associated with increased CD8^+^ tumor-infiltrating lymphocytes *in vivo*.	(107)
Chemokine silencing and CD8^+^ T cell exclusion	Human ovarian cancer; tumor-bearing mice	EZH2-mediated H3K27me3 and DNMT1-mediated DNA methylation repressed CXCL9 and CXCL10, thus limiting effector T cell trafficking.	Epigenetic reprogramming increases T-cell infiltration and improves PD-L1 blockade and adoptive T-cell transfer.	(21)
	Esophageal squamous cell carcinoma (ESCC)	Tumor-intrinsic EZH2 is associated with the immune-desert phenotype; targeting EZH2 rewired NF-κB-driven CXCL9 and reduced VEGFC to restore DC maturation.	Enhanced cytotoxic CD8^+^ T-cell infiltration	(110)
	Colon adenocarcinoma: retrospective human cohorts/computational analyses (MSKCC and TCGA) plus preclinical *in vitro* and *in vivo* models	Wild-type NCOA3 upregulated HSP90α via H3K27ac; HSP90α stabilized EZH2, leading to the deposition of H3K27me3 on the CXCL9 promoter.	SI-2 (NCOA3 inhibitor) improved the efficacy of anti-PD-L1 blockade.	(111)
	Gastric cancer (HGC-27 xenografts)	LINC00152 recruited EZH2 to CXCL9 and CXCL10 promoters; silencing promoted CXCL9/10 production and CD8^+^ T-cell infiltration.	Tumor suppression reversed by CD8^+^ T cell depletion; EZH2 silencing partly inhibited LINC00152-driven growth.	(112)
	MDS → AML (NHD13 mice; SKM-1 cells)	EZH2/H3K27me3 downregulated CXCL10 transcription and secretion, facilitating CD8^+^ T-cell exhaustion in the TME.	EZH2 inhibition (GSK126) upregulated CXCL10, decelerated MDS-to-AML transformation, and abated cell exhaustion.	(113)
	Lung adenocarcinoma (LUAD): TCGA/GEO computational analyses plus preclinical *in vitro* experiments	EZH2 expression was inversely correlated with CD8^+^ T cells, DCs, and CCL5; inhibition increased CCL5 and MHC class I without altering PD-L1.	Enhanced CD8^+^ T-cell migration and MHC-I-dependent T-cell-mediated cytotoxicity	(26)
Reawakening viral mimicry the dsRNA–STING–interferon axis	NSCLC; immunocompetent LLC model	EZH2 inhibition increased dsRNA levels and triggered IFN pathway stress dependent on TLR3/MDA5, upregulating antigen-presentation genes and PD-L1.	Tumor regression, increased CD8^+^ infiltration, and response to anti-PD-1	(89)
	Prostate cancer models	EZH2 inhibition activates a dsRNA–STING–ISG stress response that upregulates antigen presentation, Th1 chemokines, and STING-dependent PD-L1.	Increased CD8^+^ trafficking and M1 TAMs reversed PD-1 CPI resistance.	(90)
	ERα^+^ luminal breast cancer	PRC2/EZH2 silences type I IFN ligands; EZH2 inhibition activates a STAT2-dependent autocrine IFN axis.	High EZH2/low STAT2 is associated with the worst antitumor immune responses.	(91)
	Neuroendocrine (NE) SCLC; Micro-C + ATAC-seq	EZH1/2 dual inhibition (valemetostat) altered compartments, TADs, and chromatin loops, reactivating Class I MHC via enhancer-promoter looping.	Reactivated MHC class I via enhancer–promoter looping and supported a neuroendocrine-to-non-neuroendocrine lineage shift.	(114)
	PTEN-deficient glioblastoma	PTEN deficiency suppressed the ERV-MAVS-IFN axis, while 5-AZA + EZH2i reduced H3K27me3 and synergistically restored ERV activation and type I IFN signaling.	TME reprogramming boosts antitumor immunity and suppresses tumor progression	(92)
	Melanoma (B16-F10)	STING was H3K27me3-silenced; the EZH2 inhibitor induced an H3K27me3→H3K27ac shift, increasing STING, MHC-I, and chemokines.	Synergistic tumor control and increased CD8^+^ infiltration with STING agonist + EZH2i	(93)
Emerging non-canonical layers ncRNAs, circular EZH2, RNA editing, and senescence	Glioblastoma stem cells (GSCs)	DDX3-regulated circular EZH2-encoded EZH2-92aa directly repressed MICA/B and indirectly repressed ULBPs by stabilizing the EZH2.	EZH2-92aa knockdown enhanced NK cell-mediated GSC eradication and synergized with anti-PD-1 therapy.	(116)
	Pan-cancer (9,626 tumors, 32 types); immunocompetent mice	LincRNA EPIC1 interacts with EZH2 to epigenetically silence IFNGR1, TAP1/2, ERAP1/2, and MHC-I.	Genetic and pharmacological EZH2 inhibition abolished EPIC1’s immune-evasive effect and IFN-γ suppression.	(115)
	Prostate cancer	EZH2 competes with ILF2 for ADAR1 binding and reshapes A-to-I editing substrate selectivity. EZH2 depletion induces TRN1 translational repression and cytoplasmic ADAR1p110 accumulation.	ADAR1 depletion increased the sensitivity of tumors to EZH2 selective degraders.	(117)
	Pancreatic ductal adenocarcinoma (PDAC); mouse models	EZH2 epigenetically represses proinflammatory SASP genes after therapy-induced senescence.	EZH2 blockade stimulated CCL2 and CXCL9/10, enhanced NK and T cell infiltration, and drove PDAC eradication.	(118)
	Pan-cancer (including PDAC)	Overexpressed EZH2 and underexpressed RKIP form a dysregulated EZH2-RKIP axis that regulates immune evasion.	Targeting the EZH2-RKIP axis to restore immunotherapy responsiveness	(119)

## 5 EZH2 as a central regulator of the tumor microenvironment — cell-type-specific control

### 5.1 Tumor-associated macrophages: context-dependent M1/M2 reprogramming

**Table 2** summarizes the cell-type-specific effects of EZH2 perturbation across tumor-associated macrophages (TAMs), regulatory T cells (Tregs), CD8^+^ T cells, NK cells, and other myeloid populations, with an emphasis on the experimental context, perturbation strategy, and resulting immune phenotype. EZH2 occupies a paradoxical position in macrophage biology; depending on the cellular context and targeting modality, it can drive either antitumor M1 reprogramming or protumor M2 consolidation, suggesting that this duality should be a central consideration in its therapeutic exploitation (**Figure 3**). Throughout this section, the terms “M1-like” and “M2-like” are used as operational descriptions of macrophage polarization states reported in the cited experimental studies rather than as fixed macrophage lineages, because tumor-associated macrophages can occupy heterogeneous and overlapping activation states (120). Where available, we specified the experimental markers used to define these states: M1-like polarization was principally assessed by increased CD86, inducible nitric oxide synthase (iNOS), and/or tumor necrosis factor-α (TNF-α), whereas M2-like polarization was assessed by CD206 and/or arginase-1 (Arg1), with additional markers specified where reported by the original studies (121–125).

**Table 2:** Cell-type-specific effects of EZH2 perturbation in the tumor microenvironment.

Mechanistic layer	Cancer type/model	Key finding	Experimental approach	Impact on immune compartment	Ref.
Tumor-associated macrophages (TAMs) — context-dependent M1/M2 reprogramming	Colorectal cancer (CRC); 3D culture and *in vivo* co-culture	EPZ6438 increased CD86 and iNOS expression and reduced CD206 and Arg1 expression. *In vivo*, EPZ6438 reduced CD206^+^ TAMs, whereas GSK126 showed a trend toward increased CD206^+^ macrophages. Both inhibitors reduced H3K27me3 levels in the examined STAT3 promoter regions.	3D culture, co-culture of macrophages with CRC cells, ChIP-qPCR, *in vivo* CRC models	EPZ6438 promoted an M1-like shift, whereas GSK126 did not produce a comparable phenotype, and macrophage responses were inhibitor- and context-dependent.	(121)
	Glioblastoma (GBM); human/murine GBM cell co-culture with PBMC-derived macrophages/primary microglia	EZH2 suppression in GBM cells reduced TGFβ1–3 and IL-10 and elevated IL-1β and IL-6; increased M1 markers (TNFα, iNOS); and decreased M2 markers in microglia.	siEZH2, DZNep, RT-PCR, flow cytometry, phagocytosis assay, CCK8	Shifted microglia toward the M1 phenotype; restored phagocytic capacity; and reduced CD206^+^ PBMC-derived macrophages.	(122)
	Human glioblastoma specimens (n = 8); single-cell transcriptomic and correlative analysis	EZH2 was heterogeneously expressed; a TAMs subset (macrophage.3) showed high EZH2 with M2-phenotype genes and cell cycle dynamics, and subset abundance increased in GBM vs. LGG.	scRNA-seq, deconvolution analysis, IF microscopy, RT-qPCR, colony formation, CCK-8	Identified an EZH2^hi^ protumor TAM subset interacting with MES2-like malignant cells, associated with worse prognosis.	(126)
	Hepatocellular carcinoma (Hepa1–6 conditioned BMDMs)	Ezh2-deficient BMDMs exhibited enhanced M2-like polarization, stronger induction of M2-associated genes, enrichment of glycolysis and JAK/STAT pathways, and elevated pSTAT3 and Arg1 levels.	BMDM Ezh2 knockout, RNA-seq, ATAC-seq, metabolic flux analysis	Promoted M2-like macrophage polarization associated with glycolytic reprogramming in HCC-conditioned medium.	(123)
	HPV16^+^ cervical cancer (SiHa, CaSki); nude-mouse xenograft	EPZ6438 suppressed KDM6A expression in a concentration-dependent manner, and KDM6A overexpression reversed EZH2i effects on viability, IL-6/CXCL1, and p-Akt.	EPZ6438 dose-response, KDM6A rescue, THP-1/IL-4/IL-13 M2 co-culture, nude-mouse xenograft	Tumor-cell EZH2 inhibition reduced the maintenance of the experimentally induced M2-like macrophage phenotype in the conditioned environment and was associated with reduced tumor growth.	(127)
	Colorectal cancer; *in vitro* and *in vivo*	Exosomal LINC01615 transferred from CRC cells to TAMs acted as a scaffold mediating RBMX–EZH2 binding at EZH2 mRNA and promoter to amplify EZH2 expression.	Bioinformatic analysis, scRNA-seq, RIP, RNA pull-down, truncation, luciferase, ChIP, qRT-PCR, Western blot, TEM, Cre-LoxP exosome tracing	Exosomal LINC01615 induced M2 polarization of TAMs via the RBMX–EZH2 axis, promoting CRC progression.	(128)
Regulatory T cells (Tregs) — reprogramming versus destabilization	Ezh2^Y641F gain-of-function Treg model (autoimmunity)	Ezh2^Y641F Tregs displayed an effector Treg phenotype with redistributed H3K27me3, recapitulating the CD28 co-stimulation-induced expression profile and improved tissue homing.	Ezh2^Y641F mouse model, H3K27me3 ChIP-seq, transcriptome profiling, autoimmunity models	Enhanced Treg-mediated immune suppression and accelerated remission from autoimmunity	(129)
	Tumor-infiltrating Tregs (TI-Tregs); pharmacological and genetic EZH2 disruption	Disruption of EZH2 activity drove the acquisition of pro-inflammatory functions in TI-Tregs and remodeled the TME.	Pharmacological EZH2 inhibition, Foxp3-Cre genetic models	Reprogrammed TI-Tregs toward a pro-inflammatory state; enhanced CD8^+^ and CD4^+^ effector recruitment and function; less toxic than generalized Treg depletion	(94)
	Treg-specific EZH2 depletion (FoxP3^creEZH2^fl/fl); human T cells; murine tumor models	Genetic EZH2 depletion in Tregs resulted in antitumor immunity; CPI-1205 altered Treg phenotype/function and enhanced Teff cytotoxicity; ipilimumab increased EZH2 levels in peripheral T cells of treated patients.	FoxP3^creEZH2^fl/fl mice, CPI-1205, patient peripheral T cell analysis, murine tumor models	Improved antitumor responses to anti-CTLA-4 therapy	(130)
	Diffuse large B-cell lymphoma (DLBCL); 86 cases; C-NKG xenografts	EZH2 downregulated miR-9, upregulating CXCR4; CXCR4 bound exogenous CXCL12 to increase Treg differentiation; sh-EZH2 reduced intratumoral Treg infiltration.	IHC on 86 DLBCL cases, PBMC–DLBCL co-culture, sh-EZH2 C-NKG xenografts	EZH2/miR-9/CXCR4-CXCL12 axis recruits Treg infiltration; sh-EZH2 reduced Treg density and tumor viability.	(131)
	Nasopharyngeal carcinoma (NPC); 86 patients; *in vitro* aTregs and *in vivo*	LMP1 upregulated EZH2, increasing the stability of activated Tregs; DZNep depleted LMP1-HONE1 antigen-induced aTregs.	Flow cytometry, IHC, IF of 86 NPC samples, *in vitro* aTreg stability assays, *in vivo* TAA-specific T cell assays	DZNep depletion of LMP1-induced aTregs restored tumor-associated antigen-specific T cell antitumor immunity.	(132)
	Castration-resistant prostate cancer (CRPC): computational analysis of human cohorts (TCGA-PRAD and independent cohorts) plus preclinical C42-cell perturbation	Higher EZH2 expression is associated with adverse outcomes and immunosuppressive signatures; EZH2^hi^ malignant cells showed proliferative state; tazemetostat reduced H3K27me3 and upregulated TIMP3, PLCG2, SOCS3.	Bulk RNA-seq, scRNA-seq (GSE264573), NMF immune subtyping, penalized Cox, CellPhoneDB, tazemetostat C42 perturbation, RT-qPCR	EZH2 is associated with Treg-related programs and tumor-Treg crosstalk; perturbation modulates immune- and inflammation-associated genes.	(133)
CD8^+^ T cells — exhaustion, memory, and stem-like maintenance	CD8^+^ T memory precursor formation and antitumor ACT models	Ezh2 activated Id3 while silencing Id2, Prdm1, and Eomes; Akt phosphorylation of Ezh2 reduced its control of these programs; Akt-insensitive Ezh2 mutant improved memory potential.	Genetic and ACT murine models, Akt-insensitive Ezh2 mutant engineering	Akt-insensitive Ezh2 mutant CD8^+^ T cells exhibit improved memory potential and tumor control.	(135)
	Ovarian cancer; patient samples	Tumor-imposed glucose restriction sustained high levels of miR-101 and miR-26a, which constrained EZH2; EZH2 suppressed Numb and Fbxw7 via H3K27me3, activating Notch; shEZH2 elicited poor antitumor immunity.	miRNA expression analysis, shRNA EZH2 knockdown, Notch pathway analysis, patient survival	EZH2^+^CD8^+^ T cells are associated with improved patient survival, and EZH2 supports polyfunctional cytokine expression and Bcl-2 survival signaling.	(136)
	CTLs from TME; NSCLC patient cohort	miR-26a upregulated in CTLs upon TME secretome stimulation and directly targets EZH2; miR-26a-transgenic CTLs showed impaired IFN-γ and granzyme B production.	miR-26a-transgenic mice, TME secretome stimulation, NSCLC cohort validation	miR-26a inhibition increased CTL cytotoxicity and suppressed tumor growth; the miR-26a–EZH2 axis was inversely correlated in CTLs.	(137)
	Hepatocellular carcinoma (HCC): computational/single-cell analyses plus preclinical immunocompetent mouse models	A 16-gene integrated model based on autophagy, senescence, dormancy, mitochondrial, and stemness genes predicted HCC prognosis and ICI responsiveness. EZH2 is implicated in immune infiltration, activation, and HCC progression.	Integrated 16-gene model, scRNA-seq trajectory analysis, immunocompetent mouse models	EZH2 regulates CD8^+^ T cell activation and exhaustion through the MIF–CD74 signaling pathway.	(138)
	Preclinical melanoma ACT models; Pim2^-^/^-^ Pmel mice; exploratory human T-cell and CD19 CAR T-cell experiments (conference abstract)	Pim2 phosphorylates Vprbp to destabilize Ezh2. Pim2 deficiency increases Ezh2 and H3K27 methylation, inhibits autophagy, and enhances glycolysis in mouse embryonic fibroblasts.	Pim2^-^/^-^ Pmel ACT, Pim2-specific inhibitor, human T cell and CD19 CAR-T assays	Pim2^-^/^-^ CD8^+^ T cells showed enhanced effector differentiation, persistence, and melanoma control, and increased progenitor-like/memory differentiation in lymphoid organs.	(139)
Natural killer (NK) cells — maturation, cytotoxicity, and ligand recognition	Conditional Ezh2 deletion in NK lineage (Ezh2^fl/fl × Ncr1iCre); HSPC-derived human NK cells	Ezh2 deficiency increased total NK numbers and promoted terminal differentiation to the CD27^-^CD11b^+^ mature subset; direct EZH2 modulation of Pbx1.	Ncr1iCre Ezh2^fl/fl model, HSPC differentiation assays, RNA-seq, CUT&RUN-qPCR	Enhanced NK cytotoxicity with increased degranulation and IFN-γ production *in vitro* and in human HSPC-derived NK differentiation	(142)
	Ezh2-deficient NK cells; scRNA-seq	scRNA-seq identified six NK maturation stages; Ezh2 deletion caused arrest at the CD11b SP stage 5, clustered with activating-function genes.	scRNA-seq of Ezh2-deleted immature NK cells and progeny	Transcriptomic and trajectory analyses suggested an Ezh2–AP-1–Klrg1 regulatory relationship; however, a PRC2-independent mechanism was proposed but not directly established experimentally.	(143)
	Hepatocellular carcinoma (HCC) cells; NK co-culture	HCC cells showed transcriptional downregulation of NKG2D ligands; a screen of 32 epigenetic inhibitors identified EZH2 as a transcriptional repressor of NKG2D ligands.	Small-molecule and genetic EZH2 inhibition, 32-compound epigenetic inhibitor screen, NK co-culture	EZH2 inhibition enhanced NKG2D ligand-dependent NK cell-mediated eradication of HCC cells.	(144)
	Hepatic tumor; syngeneic immunocompetent mouse model	Genetic or pharmacological (GSK343) EZH2 inhibition induced CXCL10 re-expression in hepatic tumor cells, and HDAC10 was required for EZH2 recruitment to the CXCL10 promoter.	GSK343, NK cell depletion, CXCL10 rescue in immunocompetent syngeneic mice	NK cell depletion reversed the tumor-inhibitory effects of EZH2i; CXCL10 re-expression was required for tumor suppression; and stimulated NK cell migration.	(73)
	Muscle-invasive bladder cancer (KDM6A/SWI-SNF loss-of-function); xenograft	EZH2 inhibition induced immune signaling with the largest changes in IFN-γ; upregulated NK cell-associated genes MIP-1α, ICAM1, ICAM2, and CD86; and decreased ALDH2 and CK5.	EZH2 inhibitor ± cisplatin in KDM6A/SWI-SNF-mutant lines, xenograft models	Activated NK cell-related signaling drives tumor cell differentiation and cell death.	(72)
	Multiple myeloma; NK co-culture and xenografts	Exosomal NEAT1 silenced PBX1 by recruiting EZH2, and MM exosomes reduced NKG2D^+^ cells and TNFα/IFN-γ in NK cells, while NEAT1-silenced exosomes reversed these effects.	ChIP, RIP, EdU staining, CCK-8, flow cytometry, ELISA, xenograft model	Exosomal NEAT1 suppresses NK cell activity via the EZH2/PBX1 axis; NEAT1-silenced exosomes restored NKG2D, TNFα, and IFN-γ expression and inhibited tumor growth.	(145)
Myeloid-derived suppressor cells (MDSCs), dendritic cells, and neutrophils — the “dark side” of EZH2 inhibition	Tumor-bearing mice; hematopoietic progenitor cells	GSK126 treatment increased MDSCs and decreased CD4^+^ and IFN-γ^+^CD8^+^ T cells; EZH2 inhibition directs myeloid differentiation from primitive hematopoietic progenitors.	GSK126 treatment, anti-GR-1 antibody, gemcitabine/5-FU MDSC depletion	Anti-GR-1 or gemcitabine/5-FU MDSC depletion alleviated immunosuppression and restored CD4^+^/CD8^+^ infiltration and GSK126 efficacy *in vivo*.	(29)
	HNSCC mouse models; patient samples	Tazemetostat monotherapy had a limited inhibitory effect with a surge in intratumoral MDSCs; combined tazemetostat + sunitinib reduced MDSCs and Tregs.	Bioinformatics, HNSCC mouse models, tazemetostat ± sunitinib ± anti-PD-1	Combined tazemetostat and sunitinib promoted T cell infiltration, inhibited T cell exhaustion, increased intratumoral PD-L1 expression, and improved anti-PD-1 response.	(147)
	Malignant pleural mesothelioma (MPM); 3D spheroid co-culture	Prolonged tazemetostat treatment enhanced Mo-TAM recruitment and increased monocyte chemoattractant, inhibitory checkpoint, immunosuppressive, and M2-like gene expressions.	3D MPM spheroid model with monocyte recruitment and differentiation	Mo-TAMs suppressed the antiproliferative effects of EZH2 inhibition and supported tumor cell proliferation and spread.	(30)
	Pleural mesothelioma (BAP1/CDKN2A-proficient spheroids); neutrophil co-culture	EZH2 inhibition upregulated RAB27b and CD63 and increased extracellular vesicle release; EVs skewed naïve neutrophils toward the PD-L1^+^/MSLN^+^ phenotype.	Quantitative proteomic mass spectrometry, tangential flow filtration EV isolation, flow cytometry, co-culture	EV-elicited neutrophils suppressed T-cell proliferation and enhanced tumor cell growth.	(99)
	NOD.Stat5b-CA transgenic mice (autoimmune diabetes)	NOD.Stat5b-CA splenic DCs showed a tolerogenic profile with elevated PD-L1/PD-L2, increased cDC2, and decreased cDC1; increased Ezh2 and IRF4, but decreased IRF8.	NOD.Stat5b-CA transgenic model, Ezh2 inhibition, flow cytometry	Ezh2 inhibition reduced PD-L1 (not PD-L2) levels, especially in cDC2, contracted cDC2 subset, expanded cDC1, and increased pro-inflammatory cytokine production.	(148)

**Figure 3:**
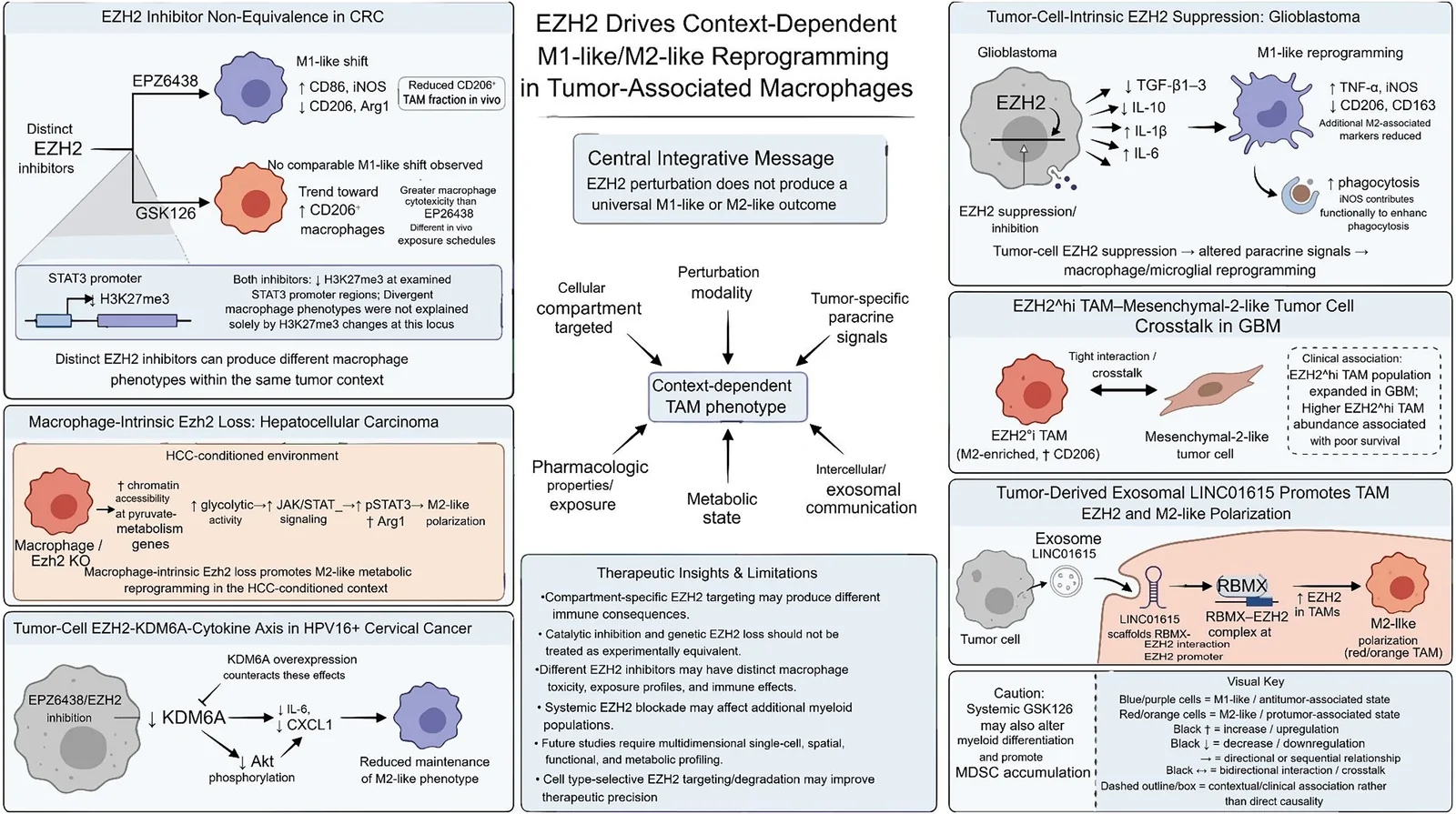
Context-dependent M1-like/M2-like reprogramming of tumor-associated macrophages by EZH2 perturbation. In colorectal cancer (CRC), EPZ6438 promotes an M1-like phenotype (↑CD86/iNOS, ↓CD206/Arg1), whereas GSK126 does not produce a comparable shift, despite both inhibitors reducing H3K27me3 at the examined STAT3 promoter regions (121). Tumor-cell EZH2 suppression in glioblastoma alters paracrine cytokine signaling, promotes TNF-α/iNOS-associated M1-like reprogramming, and enhances phagocytosis (122), whereas macrophage-intrinsic *Ezh2* loss in hepatocellular carcinoma promotes glycolytic/JAK–STAT3–Arg1-associated M2-like polarization (123). In HPV16-positive cervical cancer, tumour-cell EZH2 inhibition reduces KDM6A and IL-6/CXCL1 signaling and reduces maintenance of the experimentally induced M2-like macrophage phenotype in the conditioned environment (127). In glioblastoma, EZH2^hi M2-enriched TAMs are associated with mesenchymal-2-like tumor cells and poor survival (126), whereas CRC-derived exosomal LINC01615 promotes M2-like polarization through the RBMX–EZH2 axis (128). Collectively, macrophage responses to EZH2 perturbation depend on the targeted cellular compartment, perturbation modality, tumor-specific signaling, and metabolic context of the tumor.

In colorectal cancer models, pharmacological EZH2 inhibition produced inhibitor- and context-dependent effects on macrophage polarization rather than a uniform macrophage response (121). In tumor-conditioned macrophage cultures, EPZ6438 increased the M1-associated marker CD86 and promoted the expression of M1-associated genes, including CD86 and iNOS, while reducing M2-associated markers such as CD206 and Arg1 (121). Consistent with these *in vitro* findings, EPZ6438 reduced the proportion of CD206^+^ tumor-associated macrophages *in vivo*, whereas GSK126 did not produce the same phenotype and instead showed an increased trend in the CD206^+^ macrophage compartment (121). Both inhibitors reduced H3K27me3 at regions of the STAT3 promoter examined by ChIP-qPCR, indicating that their divergent macrophage effects cannot be explained simply by opposite effects on EZH2-dependent H3K27 methylation at this locus (121). Importantly, GSK126 exhibited greater cytotoxicity toward macrophages than EPZ6438 in this experimental system, and the two compounds were administered using different exposure schedules *in vivo*, providing potential pharmacological contributors to their divergent immune effects; however, the study did not establish a definitive molecular mechanism explaining the inhibitor-specific difference (121).

A distinct tumor-cell-intrinsic mechanism was reported in glioblastoma, where EZH2 suppression in glioblastoma cells decreased TGF-β1–3 and IL-10 levels while increasing IL-1β and IL-6 levels, thereby altering the signals encountered by co-cultured microglia and macrophages (122). In murine microglia, this intervention increased the M1-associated markers TNF-α and iNOS and decreased multiple M2-associated markers, including TGF-β1/2, STAB1, Ym1, LYVE1, Fizz1, CD206, and CD163. In human PBMC-derived macrophages, the proportion of CD206^+^ cells was also reduced (122). EZH2 suppression in glioblastoma cells also enhances microglial phagocytosis, with iNOS contributing to this effect (122). However, single-cell dissection of the same malignancy identified an EZH2^hi^ TAM subpopulation tightly interacting with mesenchymal-2-like malignant cells, enriched in M2 programs, expanded in glioblastoma (GBM) versus lower-grade glioma, and independently associated with poor survival, establishing that tumor-extrinsic, macrophage-intrinsic EZH2 enforces the protumor polarity that tumor-intrinsic EZH2 inhibition seeks to reverse (126).

The opposite direction of regulation was observed when Ezh2 was genetically deleted specifically in bone marrow-derived macrophages exposed to hepatocellular carcinoma-conditioned medium, emphasizing that macrophage-intrinsic EZH2 loss is not mechanistically equivalent to tumor-cell-directed EZH2 perturbation (123). Ezh2-deficient macrophages exhibited stronger induction of M2-associated transcriptional programs, together with increased chromatin accessibility at pyruvate-metabolism genes, enhanced glycolytic activity, and enrichment of JAK/STAT signaling (123). Phosphorylated STAT3 and the M2-associated marker Arg1 were both increased following Ezh2 loss, supporting a macrophage-intrinsic pathway in which EZH2 normally restrains HCC-induced metabolic reprogramming and M2-like polarization (123).

A further tumor-cell-dependent mechanism has been reported in HPV16-positive cervical cancer, in which EZH2 inhibition with EPZ6438 reduced KDM6A expression in SiHa and CaSki tumor cells in a concentration-dependent manner (127). This reduction in KDM6A was accompanied by decreased IL-6 and CXCL1 production and reduced Akt phosphorylation, whereas KDM6A overexpression counteracted these effects of EPZ6438, placing KDM6A downstream of EZH2 inhibition in the tumor-cell inflammatory program examined in this model (127). When macrophages were exposed to the corresponding tumor-cell-conditioned environment, EPZ6438 treatment of cervical cancer cells was associated with reduced maintenance of the experimentally induced M2 phenotype, supporting a model in which tumor-cell EZH2 sustains a KDM6A-associated inflammatory secretory program that favors M2-like macrophage polarization (127). In colorectal cancer, tumor-derived exosomal LINC01615 scaffolds the RBMX–EZH2 interaction at the EZH2 promoter to amplify EZH2 in recipient TAMs and promote M2-like polarization (128). Therefore, the apparently opposing macrophage phenotypes observed after EZH2 targeting should not be interpreted as evidence of a single bidirectional mechanism. First, the cellular compartment being perturbed differs across studies: EZH2 was suppressed in tumor cells in the glioblastoma and HPV16-positive cervical cancer models, whereas Ezh2 was genetically deleted directly in macrophages in the HCC study, and the colorectal cancer study included direct pharmacological exposure of macrophages as well as systemic treatment *in vivo* (121–123, 127). Second, genetic Ezh2 deletion and catalytic pharmacological inhibition are experimentally non-equivalent perturbations: genetic deletion eliminates the EZH2 protein, whereas catalytic inhibitors primarily interfere with its enzymatic output, making direct cross-study comparison inappropriate unless both approaches are tested within the same cellular and tumor contexts (59, 123). Third, even two catalytic EZH2 inhibitors were not interchangeable in the colorectal cancer (CRC) model: EPZ6438 and GSK126 differed in macrophage cytotoxicity, exposure schedules, *in vivo* tumor control, and their effects on CD206^+^ macrophages, despite both reducing H3K27me3 at the STAT3 promoter (121). Systemic GSK126 has also been reported to promote MDSC accumulation by altering differentiation from hematopoietic progenitors, further illustrating that whole-organism immune effects may extend beyond macrophage-intrinsic target engagement (29).

Therefore, the divergent effects of EPZ6438, GSK126, tumor-cell EZH2 suppression, and macrophage-specific Ezh2 deletion should be regarded as context-dependent experimental observations rather than interchangeable evidence for a universal M1- or M2-polarizing function of EZH2 (121–123, 127). Moreover, because macrophages in tumors occupy heterogeneous activation continua, future studies should complement conventional CD86/iNOS/TNF-α and CD206/Arg1 measurements with multidimensional single-cell, spatial, functional, and metabolic profiling (120). Collectively, these studies indicate that the consequences of EZH2 perturbation in macrophages are determined by the cellular compartment targeted, tumor-specific paracrine signals, metabolic state, and nature and duration of the perturbation rather than by EZH2 inhibition per se.

### 5.2 Regulatory T cells (Tregs): reprogramming versus destabilization

The role of EZH2 in regulatory T cell biology can be viewed not as a uniform activator of suppression, but as an epigenetic rheostat whose level of activity influences whether Tregs consolidate an effector-suppressive identity or adopt more fragile, pro-inflammatory states, a duality that may help explain why EZH2 targeting is being explored for reprogramming, rather than depleting, the intratumoral Treg compartment. Mechanistically, PRC2-deposited H3K27me3 contributes to maintaining FOXP3-dependent lineage fidelity and the transcriptional circuitry associated with T-cell receptor (TCR)/CD28-driven effector Treg (eTreg) differentiation: an *Ezh2*^Y641F gain-of-function allele redistributes H3K27me3 in naive Tregs to a pattern that prefigures CD28-activated eTreg programs, and is associated with enhanced tissue homing and suppressive capacity (129). Conversely, Treg-selective genetic or pharmacological EZH2 disruption can drive tumor-infiltrating Tregs toward pro-inflammatory features, remodeling the TME, and enabling CD8^+^/CD4^+^ effector recruitment, an effect reported to be mechanistically distinct from and potentially less autoimmunity-prone than generalized Treg depletion (94). Clinical relevance is sharpened by the observation that ipilimumab therapy elevates EZH2 levels in peripheral T cells and that CPI-1205 synergizes with anti-CTLA-4 in murine models (130), supporting the idea that EZH2 destabilization may provide a mechanistic rationale for combination checkpoint strategies.

Beyond Treg-intrinsic regulation, EZH2 appears to influence Treg accumulation through tumor-extrinsic mechanisms. In diffuse large B-cell lymphoma, tumor-cell EZH2 downregulates miR-9 to derepress CXCR4, which engages stromal CXCL12 to chemoattract Tregs; EZH2 silencing is associated with reduced intratumoral Treg density without clear changes in systemic pools (131). Viral oncoproteins may converge on similar pathways. EBV-encoded LMP1 in nasopharyngeal carcinoma induces EZH2 to stabilize activated Tregs, and DZNep treatment reduces these LMP1-dependent Tregs and is associated with the restoration of tumor-antigen-specific T cell immunity (132). Integrative bulk and single-cell transcriptomic analyses of castration-resistant prostate cancer reinforced this tumor–Treg crosstalk model, with EZH2^high malignant cells engaging in stronger inferred Treg interactions and tazemetostat inducing TIMP3/PLCG2/SOCS3 upregulation (133).

Nevertheless, these findings rest predominantly on Foxp3-Cre murine systems, co-cultures, and CellPhoneDB-inferred interactions, with limited human causal validation; genetic deletion and pharmacologic inhibition are not mechanistically equivalent, and whether intratumoral selectivity holds in humans without systemic autoimmunity remains unclear. Cell-type-selective EZH2 targeting, for example, via Treg-restricted delivery or temporally controlled degraders, may be necessary to safely translate CTLA-4 combination strategies into clinical practice.

### 5.3 CD8^+^ T: exhaustion, memory, and stem-like maintenance

EZH2’s role in CD8^+^ T cell biology appears to be neither uniformly protective nor uniformly suppressive, and a more accurate reading of the current evidence supports a context-dependent model in which PRC2/H3K27me3-mediated repression is associated with the maintenance of memory and stem-like programs under physiological conditions, yet is reshaped within the tumor microenvironment in ways associated with functional impairment (134). Mechanistically, EZH2 has been reported to silence *Id2*, *Prdm1*, and *Eomes* while sustaining *Id3*, a configuration associated with memory precursor expansion. Akt-dependent phosphorylation of EZH2 appears to attenuate this program and may bias differentiation toward terminal effectors, as Akt-insensitive EZH2 mutants have been shown to enhance memory potential in adoptive transfer models (135). In parallel, EZH2 has been reported to suppress the Notch repressors *Numb* and *Fbxw7*, which is consistent with Notch-supported polyfunctional cytokine output and Bcl-2-mediated survival in ovarian cancer models (136). Consistent with a protective T-cell-intrinsic role in this setting, the abundance of EZH2^+^CD8^+^ tumor-infiltrating T cells was associated with improved survival in the ovarian cancer cohort examined (136).

In contrast, within the tumor microenvironment, this same regulator appears to be actively constrained: glucose restriction imposed by tumor cells maintains elevated miR-101 and miR-26a, which directly target *EZH2* and suggest a metabolic–epigenetic circuit whereby nutrient scarcity may contribute to cytotoxic T-lymphocyte (CTL) dysfunction, referring to reduced effector cytokine/cytotoxic-molecule output and impaired antitumor function in the cited experimental models (136). This mirrors the findings in NSCLC, where an inverse miR-26a–EZH2 relationship is associated with impaired IFN-γ and granzyme B production in CTLs (137). Single-cell transcriptomic analysis of hepatocellular carcinoma further suggests that EZH2 may modulate the MIF–CD74 signaling axis associated with CD8^+^ activation–exhaustion transitions, although causal validation remains limited (138). Preliminary conference-reported preclinical data suggest that Pim2-mediated phosphorylation of Vprbp can promote Ezh2 destabilization; in the reported models, Pim2 deficiency was associated with increased Ezh2/H3K27 methylation, altered CD8^+^ T-cell metabolism, and improved antitumor activity in melanoma adoptive cell therapy (139).

Several limitations qualify this evidence: pharmacological and genetic EZH2 perturbations are not mechanistically equivalent; the metabolic rescue models rely on a narrow set of tumor types; the terms ‘exhaustion’ and ‘dysfunction’ are frequently conflated across these studies; and no EZH2-directed strategy has been validated clinically in adoptive cell therapy. Rather than supporting broad EZH2 inhibition in adoptive cell therapy, these findings motivate experimentally distinct strategies such as transient ex vivo EZH2 modulation during T-cell expansion (140) or genetic tuning of EZH2 activity/phosphorylation state in engineered T cells (141). Both approaches remain preclinical and have not established clinical benefit in CAR-T or TCR T cells therapy (**Figure 4**).

**Figure 4:**
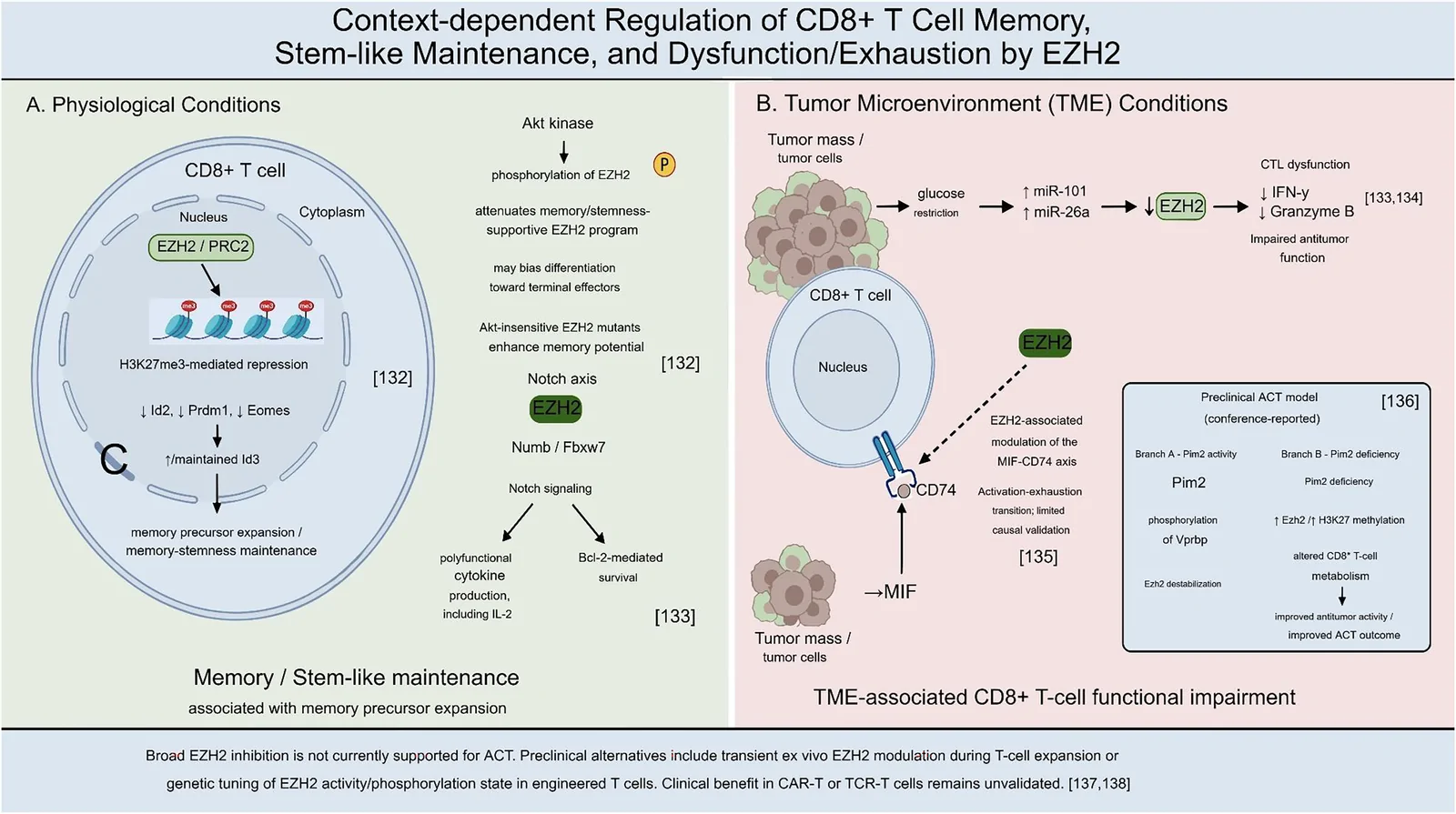
Context-dependent regulation of CD8^+^ T-cell memory, stem-like maintenance, and dysfunction by EZH2. **(A)** Under physiological conditions, EZH2/PRC2 supports memory-precursor and stem-like CD8^+^ T-cell programs through H3K27me3-dependent regulation of *Id2*, *Prdm1*, *Eomes*, and *Id3*, whereas Akt phosphorylation and Notch-associated signaling modulate memory potential, cytokine production, and survival (135, 136). **(B)** In the tumor microenvironment, glucose restriction and miR-101/miR-26a suppress EZH2 and are associated with reduced IFN-γ and granzyme B levels, whereas EZH2-associated MIF–CD74 signaling and Pim2-dependent regulation further influence CD8^+^ T-cell activation, exhaustion, metabolism, and antitumor activity (136–139). These findings support context- and modality-specific EZH2 modulation rather than broad inhibition in adoptive T cell therapy (140, 141).

### 5.4 Natural killer cells: maturation, cytotoxicity, and ligand recognition

Current evidence indicates that EZH2 operates in two spatially distinct compartments, the NK lineage itself and the tumor cell, with effects in each demonstrated in separate experimental systems. Because no cited study perturbed both compartments within a single model, concurrent dual-compartment benefit remains an inference rather than a demonstrated result (72, 73, 142–145). Within the NK lineage, conditional *Ezh2* deletion using *Ncr1*-iCre has been reported to increase total NK cell numbers and promote terminal maturation toward the CD27^-^CD11b^+^ subset, with enhanced degranulation and IFN-γ output. Mechanistic analyses suggest that this may proceed, in part, through direct modulation of the transcription factor Pbx1 (142). Evidence from NK-lineage models supports a contribution of EZH2 catalytic activity to NK-cell differentiation and function. In Study (142), NK-specific Ezh2 deletion enhanced terminal maturation, degranulation, and IFN-γ production, while pharmacological inhibition of EZH2 catalytic activity during human NK-cell differentiation produced a similar increase in mature NK-cell phenotypes and cytotoxic function. CUT&RUN-qPCR further showed reduced H3K27me3 at the Pbx1 promoter following Ezh2 loss, supporting an H3K27me3-dependent contribution to Pbx1 regulation in this setting (142). A subsequent single-cell transcriptomic study identified altered AP-1-family gene expression and NK-cell maturation trajectories after Ezh2 deletion and proposed an Ezh2–AP-1–Klrg1 regulatory axis that might involve PRC2-independent EZH2 activity (143). However, this proposed PRC2-independent mechanism was inferred primarily from transcriptomic, co-expression, and motif analyses and was not directly tested using catalytic-dead EZH2, PRC2-selective perturbation, or biochemical interaction experiments; it should therefore be regarded as hypothesis-generating rather than an established non-catalytic mechanism (143).

In contrast, tumor-intrinsic EZH2 has been reported to limit NK-cell engagement through several complementary mechanisms. In hepatocellular carcinoma, EZH2 appears to repress NKG2D ligands at the transcriptional level NKG2D ligands at the transcriptional level, and pharmacological or genetic inhibition is associated with NK-mediated eradication in a ligand-dependent manner (144). In parallel, EZH2 is recruited to the *CXCL10* promoter in an HDAC10-dependent manner, and NK cell depletion has been shown to reverse the tumor-suppressive effects of GSK343 in immunocompetent hepatic models (73). In muscle-invasive bladder cancer with KDM6A/SWI-SNF loss-of-function, EZH2 inhibition is associated with IFN-γ-driven NK-related signaling (MIP-1α, ICAM1/2, and CD86) and tumor cell differentiation (72). Tumor-derived exosomes add another layer: in multiple myeloma, exosomal NEAT1 appears to recruit EZH2 to silence *PBX1* in recipient NK cells, thereby suppressing NKG2D expression and cytokine output (145).

Notably, tumor-model heterogeneity, divergent genetic and pharmacological tools, and limited human validation temper these observations. Collectively, these observations raise the hypothesis that EZH2 inhibition could act on two compartments concurrently, permitting NK maturation while restoring NKG2D ligand display and chemokine-mediated recruitment. As noted above, no cited study tested both compartments within a single model, so this remains a hypothesis to be tested directly in NK-directed combination strategies rather than a demonstrated dual effect.

### 5.5 Myeloid-derived suppressor cells, dendritic cells, and neutrophils — the “dark side” of EZH2 inhibition

Although the preceding sections have detailed the mechanisms by which EZH2 inhibition can activate adaptive antitumor immunity, a smaller but important body of evidence indicates that the same intervention may expand or functionally skew immunosuppressive myeloid populations in specific contexts. This complicates the predominantly immunostimulatory narrative surrounding the use of EZH2 inhibitors. GSK126 treatment has been shown to expand MDSCs while reducing CD4^+^ and IFN-γ^+^CD8^+^ T-cell infiltrates, an effect attributed to EZH2-dependent redirection of myeloid differentiation from hematopoietic progenitors rather than purely tumor-cell-autonomous actions (29). These findings have been proposed as a possible contributor to the limited single-agent activity observed in the phase I trial of the clinical EZH2 inhibitor GSK2816126, which was terminated for insufficient activity (146) and align with data from HNSCC models, where tazemetostat monotherapy yielded limited tumor control accompanied by increased intratumoral MDSCs, effects partially mitigated by sunitinib co-administration (147).

Beyond MDSCs, EZH2 inhibition appears to influence other myeloid compartments in ways that may counterbalance its canonical effects. In mesothelioma spheroids, prolonged tazemetostat exposure enhances monocyte recruitment and supports differentiation toward TAM-like cells expressing inhibitory checkpoints and M2-associated molecules (30). Separately, EZH2 inhibition in BAP1/CDKN2A-proficient mesothelioma cells has been associated with RAB27b/CD63-linked upregulation of extracellular vesicle release, and brief exposure to these vesicles has been reported to skew naïve neutrophils toward a PD-L1^+^/MSLN^+^ phenotype that suppresses T-cell proliferation while supporting tumor cell growth (99). A distinct but conceptually related circuit has been described in NOD mouse dendritic cells, where a Stat5b–Ezh2 axis appears to sustain high PD-L1 expression in tolerogenic cDC2 subsets and Ezh2 inhibition selectively reduces PD-L1 (148), raising the possibility that EZH2 modulation of DC tolerance is not uniformly favorable for antitumor immunity.

Notably, these observations are largely derived from tumor-type-specific contexts (mesothelioma, HNSCC), spheroid systems, and individual inhibitors with potentially divergent off-target profiles, and human validation remains limited. Inflammation-induced emergency myelopoiesis may further complicate this interpretation. Collectively, this evidence supports the possibility that the clinical deployment of EZH2 inhibitors may necessitate combinatorial approaches that co-target the suppressive myeloid compartments. As summarized in **Table 2**, the immune consequences of EZH2 perturbation differ substantially depending on the cellular compartment, experimental model, and mode of EZH2 targeting.

## 6 Therapeutic reprogramming: synergy of EZH2 targeting with immunotherapy

Preclinical and translational strategies combining EZH2 modulation with immune checkpoint blockade, adoptive cell therapies, other epigenetic or targeted agents, and antigen- or delivery-based approaches are summarized in **Table 3**.

**Table 3:** Preclinical and translational combination strategies involving EZH2 modulation.

Therapeutic combination	Cancer type/model	Key finding	Experimental approach	Therapeutic outcome	Ref.
Combination with immune checkpoint blockade (PD-1/PD-L1/CTLA-4)	HNSCC (HPV-negative human and mouse lines; anti-PD-1-resistant syngeneic model)	EZH2 inhibition upregulated MHC class I expression and reduced H3K27me3 at the β-2-microglobulin promoter, increasing antigen-specific CD8^+^ T-cell proliferation, IFN-γ production, and tumor cell cytotoxicity.	GSK126, EPZ6438, CRISPR-Cas9 EZH2 deficiency, flow cytometry, qRT-PCR, ELISA, ChIP, anti-PD-1 combination *in vivo*	Tumor growth was suppressed by combination therapy in an anti-PD-1-resistant HNSCC model.	(100)
	Lung squamous cell carcinoma (LSCC); 2D lines, 3D murine and patient-derived organoids; autochthonous and syngeneic models	EZH2 inhibition with IFN-γ co-exposure induced MHC class I and II expression at both the mRNA and protein levels. ChIP-seq confirmed the loss of repressive marks and gain of activating marks at key loci.	EZH2 inhibitors, IFN-γ co-exposure, ChIP-seq, scRNA-seq and immune cell profiling, anti-PD-1 combination	Strong tumor control with anti-PD-1 plus EZH2 inhibition; phenotypic changes toward more tumor-suppressive immune profiles	(149)
	Triple-negative breast cancer (TNBC); *in vivo* models	Dual PI3K-mTOR inhibition with paxalisib inhibited proliferation and migration and acted upstream of both the canonical p85β-EZH2-H3K27me3 and non-canonical EZH2-NF-κB pathways.	Paxalisib, gene expression and spatial analyses	Reduced exhausted T cells, Tregs, and protumor innate populations (mast cells) overcame immunotherapy resistance and reduced primary tumor and metastasis indicators.	(150)
	TNBC	The first-in-class peptide degrader EIP103 binds to the SET domain of EZH2, induces conformational destabilization, triggers PJA2-mediated ubiquitination, and induces immunogenic cell death through lipid peroxidation.	EIP103 peptide degrader, molecular dynamics simulations, biochemical assays, TNBC models	Enhanced immune cell infiltration and antitumor immunity in TNBC models	(151)
	Oral squamous cell carcinoma (OSCC); *in vitro* and *in vivo*	Intratumoral copper depletion reduced EZH2 protein levels without a corresponding reduction in EZH2 mRNA through p62-mediated K63-linked ubiquitination associated with disruption of copper-dependent SMURF2 regulation. Higher SLC31A1 expression was associated with immunotherapy non-response in human OSCC specimens.	scRNA-seq, multiplex IHC, SLC31A1 siRNA, copper chelators, anti-PD-1 combination	Copper chelation combined with anti-PD-1 therapy produced greater tumor suppression than either treatment alone and was accompanied by increased intratumoral T-cell infiltration in a preclinical OSCC model.	(152)
Combination with adoptive cell therapies — CAR-T and TCR T cells	Human B-cell lymphoma, myeloma, AML, sarcoma, ovarian, prostate cancer preclinical models	Tazemetostat improved the efficacy of anti-CD19 CAR-T through enhanced activation, expansion, and tumor infiltration; tumors showed upregulation of adhesion, B-cell activation, inflammatory genes, and increased CAR-T avidity; valemetostat further boosted CAR-T efficacy.	Tazemetostat, valemetostat, CAR- and TCR-engineered T cell assays across multiple cancer models	Potentiated ACT in preclinical models of liquid and solid cancers by reprogramming tumors to a more immunogenic state	(35)
	DLBCL/FL syngeneic models	EZH2 inhibitors reprogrammed tumors to re-express T-cell engagement genes, reduced Tregs, promoted memory CAR CD8^+^ phenotypes, and reduced exhaustion.	Syngeneic DLBCL/FL models, intravital 2-photon imaging, CAR-T co-culture; ongoing NCT05934838, NCT05994235	Increased CAR-T recruitment and interaction within the tumor microenvironment; decreased tumor burden	(153)
	Multiple myeloma (RPMI-8226; intraosseous orthotopic xenograft)	Valemetostat + anti-BCMA CAR-T doubled complete response rate (80% vs 40%) without altering BCMA expression; RNA-seq showed upregulation of adhesion (LAMA1/2, VCAN, PECAM1), chemokines (CXCL9/10/14), and immunogenic signaling genes.	Tazemetostat, valemetostat, anti-BCMA CAR-T, teclistamab bispecific, colony formation, long-term killing, RNA-seq, orthotopic xenograft	Enhanced tumor-suppressive effect of CART-BCMA with increased serum IFN-γ; no notable toxicity; intact T-cell cytokine production.	(154)
	CD19-CAR T cells in human xenograft Raji leukemia-bearing NSG mice	CRISPR-Cas9 EZH2 ablation impaired CAR-T expansion, decreased CD45RA^-^CD62L^+^ TCM, increased TEMRA and PD-1^+^TIM3^+^ TEX cells, and failed to eliminate leukemia. EZH2^S21A/Y646N dual mutant reprogramming enabled 42.9% long-term survival.	CRISPR-Cas9 EZH2 ablation, ectopic EZH2 variant expression (WT, S21A, Y646N, S21A/Y646N), leukemia stress-test model	EZH2^S21A/Y646N-transduced CAR-T skewed away from the TEX toward the TCM phenotype, reduced AKT activity, and eliminated leukemia.	(141)
	DLBCL lines (OCI-Ly18, Toledo, SU-DHL-4); OCI-Ly18 NSG xenograft; UPenn cohort (n=69)	Tazemetostat enhanced CART19 killing with the highest effect when tumor cells were pre-incubated; increased cytokine secretion and CAR-T expansion; EZH2 activating mutation patients showed CR rate of 15.4% vs. 40.0% in wild-type.	Short-term killing assays, OCI-Ly18 xenograft, RNA-seq, zMovi avidity, scRNA-seq of circulating CART19, and retrospective human clinical-correlative cohort	Tazemetostat rewires lymphoma immunogenicity, improves tumor:CART interaction, and enriches naïve/less exhausted CAR-T cells	(155)
	B16 melanoma ACT model	Transient tazemetostat exposure prior to phenotypic exhaustion reduced H3K27 methylation, preferentially at the TCF1 promoter, in rapidly dividing T cells and preserved T-cell stemness and polyfunctionality.	Transient tazemetostat pretreatment, epigenetic reprogramming, ACT + anti-PD-1 combination	Adoptively transferred Taz-pretreated T cells exhibited superior antitumor immunity, especially when combined with anti-PD-1.	(140)
Combination with epigenetic, targeted, and cytokine-pathway agents	TNBC; 4T1 orthotopic preclinical model (conference abstract)	The CDK4/6 + EZH2 inhibitor combination activated MAVS-TBK1 via dsRNA (not dsDNA) signaling, and STAT1 phosphorylation and IFN-β secretion upregulated CXCL9/10/11.	4T1 orthotopic model, tumor-T cell co-culture, transcriptome sequencing, H151 STING inhibitor control, anti-PD-1 combination	It inhibited tumor growth, promoted CD8^+^ T cell infiltration, and synergized with anti-PD-1 monoclonal antibodies.	(156)
	T-cell lymphoma (TCL); commercial cell lines, CDX and PDX models	TCL cells developed HDAC inhibitor resistance by upregulating H3K27me3; SHR2554 + chidamide produced a synergistic effect with STAT1 identified as the key driver.	SHR2554, chidamide, cell lines, CDX, PDX, RNA-seq, ChIP-seq	Provided preclinical evidence for the SHR2554 + chidamide combination therapy strategy in TCL.	(157)
	Melanoma; cell lines, 3D spheroids, and *in vivo* models	Fenofibrate reduced EZH2 protein stability by increasing EZH2 ubiquitination and proteasome-dependent degradation, associated with increased TRIM21 and decreased OTUD4, without substantially reducing EZH2 mRNA levels.	EZH2 protein stability and ubiquitination assays; proteasome/autophagy inhibitor rescue; co-immunoprecipitation; TRIM21 knockdown and OTUD4 overexpression; GSK126/EPZ6438 combination studies.	Fenofibrate combined with GSK126 or EPZ6438 produced greater antitumor effects than either intervention alone, supporting complementary targeting of EZH2 protein abundance and catalytic activity; however, the contribution of specific non-catalytic EZH2 functions was not established.	(159)
	Ovarian high-grade serous carcinoma (HGSC); Trp53^-^/^-^ null ID8 tumors	HKMTI-1–005 induced chromatin changes that transcriptionally activated immunostimulatory gene networks, including re-expression of ERV-K endogenous retroviral elements and activation of the CXCL10-CXCR3 axis.	Dual G9A/EZH2 inhibitor HKMTI-1-005, ID8 ovarian tumor model	Increased homing of effector lymphocytes and NK cells, suppressed FoxP3^+^ CD4 + T cells, improved survival, and reduced tumor burden	(158)
	Cutaneous T-cell lymphoma (CTCL); HH and Hut78 lines; PDX models	CRISPRi screen identified an ARID1A-PRC2 synthetic-lethal axis; ruxolitinib treatment depleted ARID1A sgRNAs, indicating that JAK/STAT blockade increased CTCL dependence on EZH2.	Genome-wide CRISPRi, ruxolitinib, matrix of drug concentrations, three PDX samples	Combined ruxolitinib + EZH2 inhibitor produced strong dose-dependent synergy (Loewe synergy scores 17.54, 27.27, 20.597).	(160)
	Hepatocellular carcinoma (HCC); subcutaneous tumor model	AZD6738 had high affinity for EZH2, CCNB1, PRKDC, and PSEN1 and significantly reduced the mRNA levels of EZH2 (most pronounced), PRKDC, and CCNB1 in HCC cell lines.	Network pharmacology, scRNA-seq, spatial transcriptomics, molecular docking, RT-PCR, oral AZD6738 (75 mg/kg), flow cytometry	Enhanced immune microenvironment by promoting CD8^+^ T cell proliferation and effector-memory differentiation.	(161)
	Castration-resistant prostate cancer (CRPC); subcutaneous and spontaneous mouse models	Enzalutamide + GSK-126 synergized to reduce CRPC growth and restrained tumor neuroendocrine differentiation; antitumor activity was lost in immunodeficient mice.	Subcutaneous and spontaneous CRPC mouse models, enzalutamide + GSK-126	Awoke cytotoxic activity and IFN-γ production of tumor-specific CD8^+^ T lymphocytes	(162)
Combination with tumor-associated antigen and nanotechnology-based strategies	Ewing sarcoma	Pharmacological EZH2 inhibition at sub-cytotoxic doses selectively and reversibly induced GD2 surface expression; EZH2 bound to the promoters of two key GD2 biosynthetic enzymes, and inhibition enhanced their expression.	EZH2 inhibitor at sub-cytotoxic doses, promoter binding studies, GD2-CAR T cell co-culture	Sensitization of Ewing sarcoma cells to GD2-specific CAR gene-modified T-cell cytolysis	(163)
	Breast cancer (*in situ* mouse model)	The NP2 nanoparticle system delivered PDT and EZH2i, reversed epigenetic silence, enhanced MHC-I expression, and induced CXCL9/10 release; PDT induced ER stress and immunogenic cell death.	Polymeric nanoparticle NP2 co-delivering EZH2 inhibitor + photodynamic therapy	Synergistic immune cascade activation and anti-tumor immune response in an orthotopic breast cancer model	(165)
	Breast cancer	B-Nb_25-x_@DA@E/A nanoplatform co-delivered EZH2i and Akti, generated ROS and O_2_ locally, and alleviated tumor hypoxia.	Oxygen-defective boron-doped Nb_25-x_ nanoplatform, photothermal therapy, *in vivo* breast cancer models	Promoted dendritic cell maturation, CD8^+^ T cell infiltration, IFN-γ/Granzyme B release, and systemic antitumor immune response.	(166)
	Breast cancer	Crotonate promoted EZH2-K348 crotonylation via the ACSS2–crotonyl-CoA–p300 cascade, leading to EZH2 ubiquitination/degradation and reduced genome-wide H3K27me3 occupancy; exhibited a better metastasis-blocking effect than tazemetostat.	Crotonate, ACSS2, p300, protein crotonylation, anti-PD-L1 combination	Inhibited breast cancer growth and metastasis; crotonate + anti-PD-L1 enhanced immunotherapy response.	(167)
	TNBC; MDA-MB-231 orthotopic NSG xenograft (conference abstract)	Combination of GSK126 + A77636 (DRD1 agonist) reduced tumor weight and volume compared to vehicle and single agents; suppressed Ly6C^hi infiltration (65% decrease) and promoted Ly6C^lo accumulation.	GSK126 (2 mg/kg) + A77636 (50 mg/kg), orthotopic MDA-MB-231 NSG model, flow cytometry	Decreased monocyte populations in blood and tumors, downregulated EZH2 in monocytes/neutrophils, suppressed IL-1β, and enhanced IL-10 signaling.	(168)

### 6.1 Combination with immune checkpoint blockade (PD-1/PD-L1/CTLA-4)

Evidence from preclinical tumor systems is consistent with a model in which EZH2 perturbation is associated with shifts in tumor immunogenicity that may influence responses to checkpoint blockade; however, the underlying mechanisms appear heterogeneous rather than unified. In HNSCC, EZH2 inhibition with GSK126 or EPZ6438 has been reported to reduce H3K27me3 at the β2-microglobulin promoter, with concomitant MHC class I upregulation, increased antigen-specific CD8^+^ T cell proliferation and cytotoxicity, and tumor control in anti-PD-1-resistant models (100). A conceptually parallel observation has been made in lung squamous cell carcinoma, where IFN-γ co-exposure with EZH2 inhibition has been associated with coordinate MHC-I and MHC-II upregulation, confirmed by ChIP-seq evidence of histone-mark rebalancing, and with phenotypic shifts in single-cell immune profiles toward tumor-suppressive states (149), raising the possibility that antigen-presentation restoration is a recurring, although not universal, feature across squamous histologies. In contrast, TNBC evidence suggests that functionally relevant interventions may occur upstream of EZH2 itself: dual PI3K–mTOR blockade with paxalisib has been reported to disrupt both the canonical p85β–EZH2–H3K27me3 axis and non-catalytic EZH2–NF-κB activity, which is associated with reductions in exhausted T cells, Tregs, and protumor innate populations (150).

In addition to catalytic inhibition, several degradation strategies have been explored that may affect both enzymatic and scaffolding EZH2 functions. A multivalent peptide (EIP103) targeting the SET domain has been reported to trigger PJA2-mediated ubiquitination and immunogenic cell death through lipid peroxidation in TNBC cells (151). In oral squamous cell carcinoma (OSCC), SLC31A1 silencing or pharmacological copper chelation promotes p62-mediated K63-linked ubiquitination and autophagic degradation of EZH2 by disrupting the copper-dependent regulation of the E3 ligase SMURF2 (152). Importantly, analysis of human OSCC specimens showed that higher SLC31A1 expression was associated with non-response to immunotherapy, whereas lower SLC31A1 expression was observed in immunotherapy-sensitive tumors (152). In a syngeneic OSCC model, combining the copper chelator TEPA with anti-PD-1 therapy produced greater tumor suppression than either treatment alone, accompanied by increased intratumoral T-cell infiltration (152). However, because the clinical SLC31A1 analysis was associative and the experimental combination study did not prospectively stratify treatment efficacy according to SLC31A1 expression, high SLC31A1 expression should be described as being associated with anti-PD-1 non-response rather than as an established causal or predictive biomarker of resistance (152). Collectively, these studies support mechanistically distinct, tumor-context-dependent routes through which EZH2-directed or EZH2-modulating interventions can influence responses to immune checkpoint blockade, including the restoration of antigen presentation, remodeling of immune cell states, induction of immunogenic cell death, and post-translational depletion of EZH2 (**Figure 5**). These pathways should not be collapsed into a single “immunogenicity” mechanism because the experimentally supported intermediates and immune consequences differ across tumor models and intervention strategies (100, 149–152). Most of this evidence remains preclinical, and whether these mechanistically distinct combinations provide durable clinical benefits with PD-1/PD-L1 or CTLA-4 blockade remains to be established (100, 149–152).

**Figure 5:**
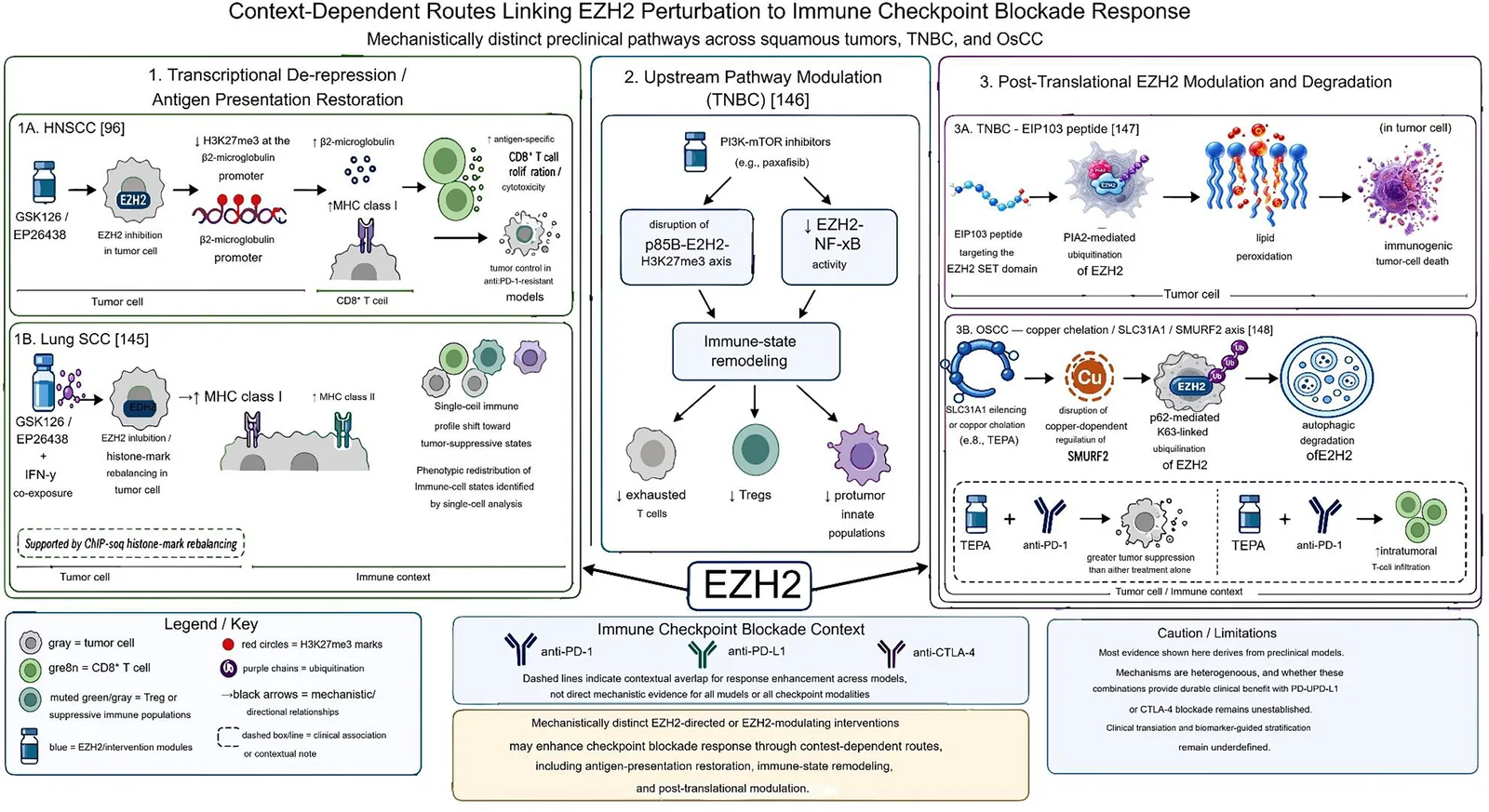
Context-dependent routes linking EZH2 perturbation to immune checkpoint blockade response. **(1A)** In HNSCC, EZH2 inhibition restores β2-microglobulin/MHC-I expression, enhances antigen-specific CD8^+^ T-cell proliferation and cytotoxicity, and improves tumor control in anti-PD-1-resistant models (100). **(1B)** In lung squamous cell carcinoma, EZH2 inhibition, together with IFN-γ, promotes MHC-I/MHC-II upregulation and shifts immune states toward tumor-suppressive phenotypes (149). **(2)** In TNBC, upstream PI3K–mTOR blockade disrupts both p85β–EZH2–H3K27me3 and EZH2–NF-κB signaling, with associated reduction of exhausted T cells, Tregs, and protumor innate populations (150). **(3A)** In TNBC, the EIP103 peptide promotes post-translational EZH2 degradation and immunogenic tumor-cell death (151). **(3B)** In OSCC, SLC31A1 silencing or copper chelation promotes SMURF2/p62-dependent autophagic EZH2 degradation and enhances anti-PD-1 activity with increased intratumoral T-cell infiltration (152). Overall, EZH2-directed interventions may enhance checkpoint blockade through mechanistically distinct, tumor-context-dependent pathways, including antigen-presentation restoration, immune-state remodeling, immunogenic cell death, and post-translational EZH2 depletion.

### 6.2 Combination with adoptive cell therapies: CAR T-cell and TCR T-cell therapies

Current evidence concerning EZH2 modulation in the context of adoptive cell therapies can be best interpreted as bifurcated across two non-aligned compartments, whose effects may be reconciled only through careful temporal and mechanistic separation. On the tumor side, EZH2 inhibition with tazemetostat or dual EZH1/EZH2 inhibition with valemetostat has been reported to reprogram lymphoma and solid tumor cells toward states associated with upregulated adhesion, chemokine, and B-cell activation genes, with increased CAR T-cell avidity and infiltration in preclinical models of human B-cell lymphoma, multiple myeloma, acute myeloid leukemia (AML), sarcoma, ovarian cancer, and prostate cancer (35). Consistent with this, syngeneic DLBCL/FL models have been associated with re-expression of T-cell engagement genes, reduced Treg frequency, and memory-skewed CD8^+^ CAR T-cell phenotypes (153). Conference-reported preclinical data on multiple myeloma have indicated that dual EZH1/EZH2 inhibition with valemetostat increases complete-response frequency when combined with anti-B-cell maturation antigen (BCMA) CAR T cells, without a corresponding increase in BCMA expression or an apparent impairment of the measured T-cell cytokine responses (154). A conference-reported retrospective analysis associated EZH2 gain-of-function mutations with lower complete-response rates following commercial CART19 therapy (155). Although this observation raises the possibility that tumor-intrinsic EZH2 status may influence CAR T-cell therapy outcomes, it remains an exploratory clinical association rather than a prospectively validated predictive biomarker.

In contrast, T-cell intrinsic evidence raises the possibility that sustained or genetic EZH2 loss may be mechanistically distinct from tumor-directed effects. Preliminary conference-reported preclinical data showed that CRISPR-mediated EZH2 ablation in CD19 CAR T cells was associated with impaired expansion, reduced central memory phenotypes, and increased PD-1^+^TIM-3^+^ populations, with AKT hyperactivation implicated in the observed phenotype (141). In the same study, reconstitution with the AKT-phosphorylation-resistant/gain-of-function EZH2^S21A/Y646N variant was associated with improved tumor control (141). (EZH2^Y646N corresponds to the Y641 residue under the 746-amino-acid isoform numbering used in Section 2.1; both designations refer to the same SET-domain tyrosine.) This is partially reconciled by findings suggesting that transient tazemetostat exposure during ex vivo expansion may reduce H3K27me3 at the TCF7 (TCF1) promoter and preserve stemness without the detrimental effects of sustained loss (140).

Collectively, these observations support a model in which tumor-directed EZH2 inhibition may influence immunogenicity, whereas T-cell intrinsic effects appear to be time-dependent. Most data are derived from xenograft and syngeneic murine systems with limited human validation, and genetic deletion is not mechanistically equivalent to pharmacological inhibition of the target. These considerations may inform engineering strategies, including transient ex vivo exposure, AKT-insensitive EZH2 variants, and cell-compartment-selective delivery, although their clinical translation remains to be determined.

### 6.3 Combination with epigenetic, targeted, and cytokine-pathway agents

As outlined in Section 2.2, EZH2 operates within an interconnected epigenetic network rather than as an isolated therapeutic target; accordingly, the combinations discussed below should be interpreted according to the specific chromatin dependency being perturbed rather than as interchangeable strategies for intensifying EZH2 inhibition (21, 45, 69–72, 75, 76). In conference-reported preclinical TNBC models, combined CDK4/6 and EZH2 inhibition was associated with the activation of dsRNA-dependent MAVS–TBK1 signaling, STAT1 phosphorylation, IFN-β production, and increased CXCL9/CXCL10/CXCL11 expression; these changes were accompanied by increased CD8^+^ T-cell recruitment and enhanced activity of anti-PD-1 treatment in the reported experimental models (156). A distinct but partially overlapping epigenetic–epigenetic interaction has been described in T-cell lymphoma, where upregulated H3K27me3 is associated with HDAC-inhibitor resistance. The EZH2 inhibitor SHR2554 combined with chidamide has been reported to produce transcriptional changes centered on STAT1, which were identified using RNA-seq and ChIP-seq (157). In contrast, dual G9A/EZH2 inhibition with HKMTI-1–005 in ovarian high-grade serous carcinoma has been associated with chromatin reorganization, re-expression of ERV-K retroviral elements, activation of the CXCL10–CXCR3 axis, and suppression of FoxP3^+^ CD4 T cells (158), an outcome that appears mechanistically related to, yet distinct from, the MAVS-driven circuit in TNBC. In melanoma models, fenofibrate reduced EZH2 protein abundance without a corresponding reduction in EZH2 mRNA, indicating post-translational rather than transcriptional regulation of EZH2 (159). Cycloheximide-chase and pharmacological rescue experiments showed that fenofibrate accelerated EZH2 protein turnover and that proteasome inhibitors reversed this reduction, supporting ubiquitin–proteasome-dependent degradation rather than autophagic degradation (159). Mechanistically, fenofibrate increased EZH2 ubiquitination by increasing the levels of E3 ubiquitin ligase TRIM21 and decreasing the levels of deubiquitinase OTUD4. It also increased the association between EZH2 and TRIM21 and reduced the association between EZH2 and OTUD4 (159). Thus, in this context, “EZH2 destabilization” specifically refers to reduced EZH2 protein stability resulting from enhanced ubiquitination and subsequent proteasomal degradation, rather than simply inhibiting its methyltransferase activity (159). Therefore, the interaction with catalytic EZH2 inhibitors should be distinguished from a direct increase in inhibitor potency. GSK126 and EPZ6438 target EZH2 methyltransferase activity, whereas fenofibrate reduces cellular EZH2 protein abundance through enhanced ubiquitin-dependent degradation (159). Experimentally, combining fenofibrate with either GSK126 or EPZ6438 produced greater inhibition of melanoma cell proliferation and three-dimensional tumor spheroid viability than the respective single-agent treatments, and enhanced tumor suppression was also observed *in vivo* (159). These findings support a complementary therapeutic model in which catalytic inhibition of EZH2 is combined with the reduction of EZH2 protein abundance, thereby extending EZH2 targeting beyond the inhibition of its methyltransferase activity alone (159). However, although depletion of EZH2 protein could theoretically affect functions that are retained during catalytic inhibition, Study (159) did not experimentally isolate the contribution of individual non-catalytic EZH2 functions to the observed combination effect; therefore, this mechanism should not be presented as the established cause of therapeutic synergy (159).

Signaling pathway combinations raise different mechanistic considerations. Preliminary conference-reported preclinical data from cutaneous T-cell lymphoma identified an ARID1A–PRC2 vulnerability through genome-wide CRISPR interference screening and suggested that JAK/STAT blockade may increase dependence on EZH2. Combined ruxolitinib and EZH2 inhibition also showed dose-dependent activity in patient-derived xenograft models (160). In a mixed computational and preclinical study of hepatocellular carcinoma, analyses incorporating network pharmacology, single-cell/spatial transcriptomics, molecular docking, and experimental validation associated AZD6738 treatment with reduced EZH2 transcript levels and changes in CD8^+^ effector-memory differentiation (161). These findings suggest a possible DNA damage response–EZH2 interface but do not establish EZH2 as the direct mediator of the immunologic effects of ATR inhibition. Another axis has been described in castration-resistant prostate cancer, where enzalutamide combined with GSK126 has been reported to restrain neuroendocrine differentiation and support CD8^+^ IFN-γ production, with activity lost in immunodeficient mice (162), consistent with an immune-dependent effect.

Collectively, these observations were derived largely from xenograft, syngeneic, and *in vitro* systems employing mechanistically divergent inhibitors (GSK126, SHR2554, HKMTI-1-005, and AZD6738) and spanned heterogeneous tumor contexts. The dominant pathway in any given setting, whether it is interferon, chromatin accessibility, replication stress, or hormonal, remains to be determined, and human validation is limited. These combinations warrant further investigation in biomarker-stratified settings.

### 6.4 Combination with tumor-associated antigen and nanotechnology-based strategies

Emerging efforts to integrate EZH2 modulation with antigen-directed, nanotechnological, and non-classical signaling approaches are best understood as a set of mechanistically distinct strategies rather than unified strategies, each addressing a different limitation of conventional epigenetic–immune combinations. In Ewing sarcoma, EZH2 was shown to occupy the promoters of two GD2 biosynthetic enzymes, and pharmacological EZH2 inhibition at sub-cytotoxic doses de-repressed these genes, producing selective and reversible surface GD2 re-expression and increased susceptibility to GD2-directed CAR T-cell cytolysis (163). This observation is conceptually distinct from, though potentially complementary to, studies linking EZH2 inhibition to MHC-I restoration, in that the target in question is a ganglioside with heterogeneous baseline expression rather than a conventional antigen-processing machinery component. In medulloblastoma, a complementary translational/preclinical study found that tazemetostat increased GD2 expression in GD2-low tumor cells and enhanced their susceptibility to GD2-directed CAR T-cell cytotoxicity, providing a mechanistic rationale for antigen upregulation before CAR T-cell targeting rather than evidence of a clinically validated EZH2–CAR T-cell combination therapy (164).

A separate line of investigation has explored spatially controlled delivery using nanotechnological platforms. A photodynamic polymeric nanoparticle system co-delivering an EZH2 inhibitor has been reported to associate reactive oxygen species–driven endoplasmic reticulum stress with features of immunogenic cell death, together with MHC-I upregulation and CXCL9/10 release in an orthotopic breast cancer model (165). In a related but mechanistically different design, a boron-doped oxygen-defective Nb_25-x_ platform co-delivering EZH2 and Akt inhibitors was associated with photothermal tumor cell disruption, oxygen generation, dendritic cell maturation, and CD8^+^ T cell infiltration in experimental settings (166). These approaches attempt to address delivery and localization rather than antigen availability per se.

Post-translational and receptor-mediated strategies represent a different conceptual framework. Crotonate modifies EZH2 at Lys348 through an ACSS2–crotonyl-CoA–p300 cascade, with K348 crotonylation associated with EZH2 ubiquitination, reduced genome-wide H3K27me3 occupancy, and an additive interaction with anti-PD-L1 in breast cancer (167). Preliminary conference-reported preclinical data from an orthotopic TNBC model associated combined GSK126 and dopamine D1 receptor agonism with reduced Ly6C^hi monocyte infiltration and changes in IL-1β/IL-10-associated signaling (168). Several caveats temper this landscape. The reversibility and heterogeneity of EZH2 inhibitor-induced GD2 re-expression raise the possibility of antigen escape under sustained CAR T-cell pressure; nanotechnological platforms remain constrained by reproducible biodistribution and light-penetration limits; and post-translational or receptor-level strategies depend heavily on cellular metabolic state and systemic physiology, with undefined therapeutic windows.

Collectively, these observations are largely derived from *in vitro*, spheroid, and xenograft systems, rely on delivery platforms with uncertain pharmacokinetics, and involve antigens (notably GD2) with known heterogeneity, and human validation is lacking. These heterogeneous approaches may provide a framework for further investigation, although their clinical applicability remains undetermined.

## 7 Clinical translation: human evidence and early-phase trials

The mechanistic and preclinical rationale for combining EZH2-directed therapy with immunotherapy has entered early clinical evaluation; however, the available prospective human evidence remains limited to small, predominantly non-randomized, early phase studies (101–103). Accordingly, this section is restricted to prospective interventional human studies and trial-associated correlative analyses; retrospective human biomarker studies, computational analyses, and preclinical experiments are discussed separately in the mechanistically relevant sections of this review. Selected active or ongoing EZH2- or EZH1/EZH2-directed immunotherapy combination trials are summarized in **Table 4**.

**Table 4:** Selected active or ongoing clinical studies of EZH2- or EZH1/EZH2-directed therapy combined with immunotherapy (registry status as of August 2026).

Trial/phase/status	Disease setting	EZH-directed regimen	Key clinical design/objective	Biomarker or correlative strategy	Ref.
NCT03854474 Phase Ib/II Active, not recruiting	Locally advanced or metastatic urothelial carcinoma	Tazemetostat + pembrolizumab	Pilot combination study evaluating the clinical activity and safety of EZH2 plus PD-1 inhibition in advanced urothelial carcinoma.	The protocol-specified correlative analyses include tumor EZH2 and H3K27me3 in relation to response, alterations in histone-methylation genes, and longitudinal immune analyses, including T-cell phenotypes/clonality, tumor-infiltrating lymphocytes, and neoantigen profiling.	(186)
NCT05353439 Phase I Active, not recruiting	Recurrent small-cell lung cancer (platinum-sensitive and platinum-resistant disease eligible)	Tazemetostat + topotecan + pembrolizumab	Dose-escalation/expansion studies. The primary objectives included MTD, RP2D (incorporating pharmacodynamic parameters, efficacy, and tolerability), and safety. The preliminary ORR, PFS, DOR, and OS were secondary objectives.	Exploratory profiling includes RNA-seq, modulation of EZH2 targets including SLFN11 and MHC, candidate predictive biomarkers, DNA/RNA-based mechanisms of sensitivity or resistance, and circulating cell-free DNA.	(187)
NCT05934838 Phase I Active, not recruiting	Previously treated DLBCL, follicular lymphoma, or mantle cell lymphoma receiving standard-of-care CD19 CAR T-cell therapy	Tazemetostat given before/around and after standard-of-care CAR T-cell therapy	The feasibility and safety of administering tazemetostat around CAR T-cell therapy, including the feasibility of CAR T-cell manufacturing, response, and survival endpoints, were secondary.	Exploratory studies included immune profiling of peripheral blood and CAR T-cell products and assessment of CAR T-cell expansion. Preliminary correlative findings have been reported in a 2025 Blood meeting abstract.	(102, 188)
NCT05994235 Phase II Active, not recruiting	Previously untreated follicular lymphoma	Tazemetostat + mosunetuzumab (CD20×CD3 bispecific antibody)	Single-arm study evaluating the safety and efficacy of a front-line combination	The public protocol specifies correlative studies before and after tazemetostat treatment and serial peripheral-blood collections around mosunetuzumab treatment; however, the public record reviewed does not specify a validated predictive biomarker for the combination.	(189)
NCT06242834 Phase II Active, not recruiting	Aggressive B-cell non-Hodgkin lymphoma following autologous stem-cell transplantation or CAR T-cell therapy	Tazemetostat + pembrolizumab	Single-group maintenance study; the primary objective was event-free survival with 12 months of combination maintenance. PFS and OS were the secondary objectives.	Exploratory objectives include changes in immune cell subsets and cytokines measured by flow-based assays and proteomics.	(190)
NCT04388852 Phase Ib Active, not recruiting	Metastatic prostate, urothelial, and renal cell cancers	Valemetostat (DS-3201; dual EZH1/EZH2 inhibitor) + ipilimumab	Dose-finding/safety study of dual EZH1/EZH2 inhibition with CTLA-4 blockade	The protocol-specified secondary objectives include immunological and molecular effects in tissue samples and assessment of treatment-related changes in the tumor microenvironment.	(191)
NCT06644768 (KEYNOTE-F85) Phase Ib/II Active, not recruiting	First-line advanced/metastatic NSCLC without actionable genomic alterations; PD-L1 TPS ≥50%	Valemetostat + pembrolizumab; phase II includes pembrolizumab-alone control	Phase Ib evaluated safety and established the valemetostat RP2D; phase II prospectively compared the combination with pembrolizumab alone.	PD-L1 TPS ≥50% is an eligibility biomarker; central 22C3 pharmDx testing is used when PD-L1 testing is not the standard of care. However, this is not an EZH2-specific predictive biomarker.	(192)
NCT07717242 (SYSTEM) Phase II Recruiting	HER2-positive cT2–4aN0M0 muscle-invasive urothelial carcinoma of the bladder	Zeprumetostat (SHR2554) + disitamab vedotin + toripalimab (Arm B of a four-arm neoadjuvant study)	Multicenter, open-label, non-randomized four-arm study of sensitization strategies added to disitamab vedotin plus PD-1 blockade. Primary endpoint: clinical complete response after six neoadjuvant cycles and cTURBT assessment.	HER2 expression ≥1+ by pretreatment tumor immunohistochemistry is required for eligibility. The protocol also includes exploratory biomarker analyses; zeprumetostat regimen is only one of four experimental arms.	(193)
NCT07555470 (CUREFL03) Phase II Recruiting	Follicular lymphoma: previously untreated high-risk, previously untreated low-tumor-burden, and relapsed/refractory cohorts	Zeprumetostat (SHR2554) + mosunetuzumab (CD20×CD3 bispecific antibody)	Prospective, multicenter, open-label, multi-cohort phase II study with an initial safety run-in followed by cohort expansion; target enrollment: 80.	The public registry record reviewed did not specify an EZH2-specific validated predictive biomarker; cohorts were defined by clinical disease setting and risk.	(194)

Registry status reflects the public clinical trial record as of August 2026 and should not be interpreted as confirmation of ongoing recruitment or continued study-drug administration. Tazemetostat (Tazverik) was voluntarily withdrawn by Ipsen from all indications and all Ipsen markets on March 9, 2026; the FDA subsequently withdrew approval of Tazverik in the United States effective June 22, 2026.

The clinical landscape changed substantially in 2026. On March 9, 2026, Ipsen announced the voluntary withdrawal of Tazverik (tazemetostat) from all indications and all Ipsen markets following emerging safety data from the confirmatory SYMPHONY-1 trial concerning secondary hematologic malignancies (169). The U.S. FDA subsequently withdrew approval of the Tazverik new drug application (NDA), encompassing its accelerated-approval indications, effective June 22, 2026 (170). The tazemetostat studies discussed below should therefore be interpreted as previously reported clinical evidence rather than as evidence of the drug’s current regulatory availability. EZH2- and EZH1/EZH2-directed therapy nonetheless remains a licensed therapeutic class: valemetostat retains Japanese approvals in relapsed/refractory adult T-cell leukemia/lymphoma and peripheral T-cell lymphoma, and zeprumetostat (SHR2554) holds conditional approval in China for relapsed/refractory peripheral T-cell lymphoma. Whether the hematologic safety signal observed with tazemetostat represents a class effect of sustained systemic H3K27me3 suppression or an agent- or regimen-specific finding is currently unresolved and requires dedicated long-term hematologic follow-up of these agents.

### 7.1 Clinical experience combining EZH2 inhibition with immune checkpoint blockade

Published early phase studies of EZH2 inhibition combined with immune checkpoint blockade remain small and non-randomized, and their results currently support feasibility and safety characterization rather than establishing incremental efficacy over checkpoint blockade alone (101, 103). In an open-label phase Ib study of tazemetostat plus atezolizumab in 43 patients with relapsed/refractory DLBCL, the recommended phase II dose (RP2D) consisted of tazemetostat 800 mg twice daily plus atezolizumab 1200 mg intravenously every three weeks (103). At the RP2D, the most frequently reported adverse events occurring in ≥20% of patients were anemia (26%), fatigue (23%), and nausea (23%) (103). At this dose level, the objective response rate was 16%, including a complete response rate of 7%, while the median progression-free survival and overall survival were 2 and 13 months, respectively (103). Exploratory molecular assessment included EZH2 mutation status, and responses were observed among a small number of patients whose tumors harbored EZH2 mutations; however, the number of mutation-positive patients was insufficient to establish EZH2 mutation as a predictive biomarker for benefit from the combination (103).

A subsequent phase I dose-escalation study enrolled 12 patients with recurrent or metastatic HNSCC and evaluated tazemetostat at 400, 600, and 800 mg twice daily together with pembrolizumab 200 mg; tazemetostat 800 mg twice daily was selected as the recommended phase II dose (101). Among the nine patients evaluable for dose-limiting toxicity, no dose-limiting toxicity occurred during cycle 1; across all 12 treated patients, the most frequent adverse events were anemia in 10 patients (83%), fatigue in eight (67%), hyponatremia in seven (58%), elevated alkaline phosphatase in seven (58%), and dry skin in five (42%) (101). No grade 4–5 treatment-related adverse events occurred, while grade 3 treatment-related anemia occurred in two patients; no immune-related adverse event requiring corticosteroid treatment was reported. No objective responses were observed; five of 12 patients (42%) achieved stable disease and seven (58%) had progressive disease as their best response, with a median stable-disease duration of 2.1 months (range, 0.8–4.4 months) (101). Two features of this study constrain its interpretation: eligibility was restricted to patients with disease refractory to prior pembrolizumab or nivolumab, a population in which the expected response rate to re-challenge is low irrespective of the added agent; and the study was terminated before completing its planned phase 2 component. The absence of objective responses should therefore be read as consistent with feasibility-stage data in an anti-PD-1-refractory cohort rather than as a definitive negative efficacy result (101, 171). The published phase I report did not establish a relationship between clinical outcome and a tumor EZH2/PRC2 activity biomarker, H3K27me3 target engagement, or another prospectively validated EZH2-related predictive marker; therefore, the study cannot determine whether a biomarker-defined subgroup derived preferential benefit from the combination (101). Additional conference-reported data are available from ETCTN 10183 (NCT03854474), a phase I/II study of tazemetostat 800 mg twice daily plus pembrolizumab 200 mg every three weeks in advanced urothelial carcinoma (172). In the 2024 report, treatment-related grade 3–4 adverse events occurred in eight patients, with anemia and lymphopenia the most frequent high-grade events (two patients each); preliminary antitumor activity was reported, but the investigators characterized any improvement over historical pembrolizumab outcomes as modest (172). The trial incorporated translational analyses of COMPASS-related alterations and EZH2 activity, but the reported data have not yet established a validated molecular predictor of response to the combination (172).

Taken together, these studies demonstrate that tazemetostat can be co-administered with PD-1/PD-L1 blockade at the evaluated doses; however, the modest response rates, small sample sizes, absence of randomized comparator arms, and limited biomarker-response analyses preclude conclusions regarding additive efficacy or predictive patient selection (101, 103).

### 7.2 Clinical translation of EZH2 inhibition with CAR T-cell therapy

The preclinical rationale for combining tumor-directed EZH2 inhibition with adoptive cellular therapy has entered early clinical evaluation in B-cell lymphomas, although the currently available results from this study are conference-reported (102). Preliminary conference-reported results from a single-center phase Ib study described 13 heavily pretreated patients with DLBCL (n = 7), follicular lymphoma (n = 5), or mantle cell lymphoma (n = 1) who received tazemetostat before and around standard-of-care CD19-directed CAR T-cell therapy, and CAR T-cell manufacturing was successfully completed for all enrolled patients (102). Cytokine release syndrome (CRS) occurred in 10 patients (77%), including one grade 3 event, whereas immune effector cell-associated neurotoxicity syndrome (ICANS) occurred in six patients (46%) and was limited to grade 1–2 events (102). Among patients with DLBCL, five of seven achieved a complete response, all five patients with follicular lymphoma achieved complete responses, while the single patient with mantle cell lymphoma achieved a partial response (102). At a median follow-up of 11 months, 54% of the cohort remained progression-free and 77% were alive. Correlative immune profiling showed an increase in naïve CD8^+^ T cells and memory-to-effector ratios, along with a reduction in terminally differentiated T-cell populations, after initiation of tazemetostat (102). These correlative changes should not be interpreted as demonstrating that tazemetostat caused the observed clinical responses because the study was small, single-arm, lacked a control group receiving CAR T-cell therapy without tazemetostat, and had a relatively short follow-up. Accordingly, these preliminary conference-reported human data support the feasibility of administering tazemetostat around CAR T-cell therapy; however, controlled and fully reported studies are required to determine whether EZH2 inhibition improves response depth, durability, or CAR T-cell persistence beyond that expected with CAR T-cell therapy alone (102).

### 7.3 Early clinical development of other EZH2 inhibitors and remaining translational gaps

Conference-reported early phase monotherapy data provide additional preliminary information on the clinical tractability and toxicity profile of EZH2 inhibition but do not provide evidence of synergy with immunotherapy (173). In preliminary phase I dose-escalation data reported at a scientific meeting, 20 patients with relapsed/refractory non-Hodgkin lymphoma or advanced solid tumors were enrolled in the TR115 study at the reported data cut-off (173). The most common treatment-related adverse events were thrombocytopenia (50%; grade ≥3, 15%), leukopenia (45%; grade ≥3, 10%), anemia (40%; grade ≥3, 15%), and neutropenia (35%; grade ≥3, 10%) (173). Among 11 response-evaluable patients with non-Hodgkin lymphoma, the reported objective response rate was 63.6% and the disease-control rate was 81.8%. All six evaluable patients with peripheral T-cell lymphoma responded, although the very small subgroup and conference-report format precluded reliable estimation of subtype-specific efficacy (173). These preliminary findings support further clinical evaluation of TR115 but should not be extrapolated to immunotherapy combinations because the reported study evaluated TR115 as a monotherapy, and the available results have not been presented as a full peer-reviewed clinical report (173). Taken together, current human evidence shows that pharmacological EZH2 inhibition is clinically feasible and can be combined with checkpoint blockade or CAR T-cell therapy; however, evidence for incremental immunotherapeutic efficacy remains insufficient because the available combination studies are early phase, non-randomized, and limited in sample size (101–103). Therefore, prospective randomized evaluations, longer follow-ups, and biomarker-defined patient selections are required to determine whether the mechanistic effects of EZH2 modulation described in preclinical systems translate into clinically meaningful improvements in immunotherapy outcomes (101–103).

## 8 Challenges, context-dependent roles, and resistance mechanisms

The preceding sections describe both immunostimulatory and immunosuppressive consequences of EZH2 perturbation across tumor and immune compartments. Rather than reiterating these mechanisms, this section focuses on the principal barriers that may limit durable therapeutic benefit: compartment-specific immune liabilities, exposure- and timing-dependent effects on effector cells, and adaptive tumor responses to EZH2 perturbation (29, 31, 123, 174–177).

### 8.1 Compartment-specific liabilities and the therapeutic window of EZH2 inhibition

As discussed in Sections 5.1 and 5.5, EZH2 perturbation can produce unfavorable immune effects in selected preclinical contexts, including MDSC expansion after systemic GSK126 exposure, M2-like myeloid reprogramming, and treatment-associated changes in monocyte or neutrophil states (29, 30, 99, 123). These findings are not repeated here in detail, but collectively establish that the tumor-directed benefits of EZH2 inhibition may coexist with opposing effects in non-malignant immune compartments.

A distinct liability has been demonstrated in activated CD8^+^ T cells. In CT26 tumor models, EZH2 inhibition impaired the antitumor efficacy of agonistic 4-1BB therapy and was associated with reduced CD8^+^ T-cell accumulation, survival, and effector programming (31). Mechanistically, EZH2 inhibition increased the pro-apoptotic protein BIM in activated CD8^+^ T cells, providing evidence that the disruption of T-cell-intrinsic EZH2 activity can compromise survival during 4-1BB-driven expansion (31). This finding is important because it demonstrates that an EZH2-targeted intervention that is advantageous in tumor cells cannot be assumed to be immunologically favorable when the same drug simultaneously reaches activated effector T cells (31, 100).

These observations define a therapeutic window problem rather than a uniform contraindication to EZH2 targeting: the net effect of treatment likely depends on which cellular compartments are exposed, the duration and intensity of EZH2 suppression, and the immune mechanism required for the accompanying therapy. Importantly, current studies do not establish a general exposure threshold at which tumor-cell epigenetic reprogramming can be preserved while deleterious effects on myeloid or lymphoid compartments are avoided (29, 31, 140). This uncertainty argues against treating all EZH2 inhibitors, genetic perturbations, dosing schedules, and cellular compartments as mechanistically interchangeable and identifies cell-selective delivery, pharmacodynamic monitoring, and schedule optimization as unresolved translational challenges rather than established solutions (29, 31, 121, 140). A further safety concern emerged from the clinical development of tazemetostat. In the randomized, double-blind confirmatory SYMPHONY-1 trial (tazemetostat versus placebo, each added to lenalidomide plus rituximab, in relapsed/refractory follicular lymphoma), hematologic second primary malignancies were reported in 18 of 318 patients (5.7%) in the tazemetostat arm as of 6 March 2026, compared with no reported events in the control arm; three patients died and 14 had unresolved events at that data cut-off (171). The majority of these events were myelodysplastic syndrome and acute myeloid leukemia, with B-cell acute lymphoblastic leukemia and clonal cytopenia of undetermined significance also reported (171). This substantially exceeded the approximately 1.7% incidence recognized at the time of accelerated approval and prompted voluntary withdrawal of Tazverik following reassessment of its risk–benefit profile (169–171). The clinical observation is nonetheless biologically coherent: although EZH2 frequently functions as an oncogenic driver in several malignancies, loss-of-function *EZH2* alterations have been recurrently identified in myeloid neoplasms, including MDS and MDS/MPN, supporting a tumor-suppressive role for EZH2 in specific hematopoietic contexts (178–180). Because these events arose in the tazemetostat-containing arm of a randomized, double-blind, active-controlled trial with no corresponding events in the control arm, the imbalance is unlikely to be attributable to the lenalidomide–rituximab backbone or to the underlying disease alone. What remains unestablished is the proximate mechanism, specifically, whether these events reflect on-target inhibition of EZH2 catalytic activity in hematopoietic progenitors, an interaction specific to the combination regimen, or agent-specific properties of tazemetostat, and therefore whether the signal extends to other selective EZH2 or dual EZH1/EZH2 inhibitors. The clinical observation is nonetheless biologically coherent, because loss-of-function *EZH2* alterations have been recurrently identified in myeloid neoplasms, including MDS and MDS/MPN, supporting a tumor-suppressive role for EZH2 in specific hematopoietic contexts (178–180). A mechanistically distinct consideration concerns the B-cell compartment itself. In mouse CH12F3 and human MEC-1 B-cell models, EZH2 inhibitor monotherapy did not significantly change AID expression or the frequency of AID-dependent chromosomal translocations (181). In contrast, combining EZH2 inhibition with phosphoinositide 3-kinase δ (PI3Kδ) inhibition, which increases AID expression, significantly increased translocation frequency relative to either agent alone in both models, and EZH2 inhibition further increased translocation formation in DNA ligase IV (Ligase4)-deficient mouse B cells (181). Mechanistically, EZH2 inhibition in B cells depleted H3K27me3 while concurrently increasing H3K27ac, increasing transcriptional activity and thereby facilitating translocation formation in the presence of high AID activity or Ligase4 deficiency (181). The authors interpreted these findings as supporting the genetic safety of EZH2 inhibitors as monotherapy in these models, while indicating an increased risk of genomic instability in B cells predisposed to translocation, such as those with high AID levels or impaired DNA repair (181).

Whether this mechanism bears on the clinical signal described above is a hypothesis raised here rather than a conclusion of the original study, which was reported before the SYMPHONY-1 safety data became available. Three constraints apply. First, the permissive interaction was demonstrated with PI3Kδ co-inhibition rather than with the lenalidomide–rituximab backbone used in SYMPHONY-1. Second, AID-dependent translocation formation is a property of activated mature B cells and would not be expected to account for the myelodysplastic syndrome and acute myeloid leukemia events that predominated in that trial; its relationship to the single B-lineage entity reported, B-cell acute lymphoblastic leukemia, is likewise uncertain, since precursor-B cells are not characteristically AID-high. Third, the preclinical monotherapy finding concerns AID-dependent translocation formation in cell-based models and does not address the incidence of second primary malignancies observed clinically with tazemetostat (169–171, 181).

Loss-of-function EZH2 biology in the myeloid compartment and AID-dependent genomic instability in the B-cell compartment are therefore best regarded as complementary rather than competing considerations, since they concern different lineages, and neither can presently be advanced as an explanation of the clinical signal without direct testing in patient-derived material (171, 178–181). Prolonged systemic EZH2 suppression therefore warrants careful hematologic evaluation, including lineage-resolved assessment of emerging clonal hematopoiesis given the clonal cytopenia of undetermined significance reported in this setting, particularly for preventive or long-term interception strategies in which chronic exposure of hematopoietic compartments would be required (171).

### 8.2 Adaptive resistance and treatment-design constraints

Beyond compartment-specific immune liabilities, the durability of EZH2-directed therapy may also be limited by treatment-exposure effects and adaptive tumor evolution. Preliminary conference-reported data indicate that prolonged tazemetostat exposure did not substantially impair the initial cytotoxic activity of CD19-directed CAR T cells but reduced sustained serial tumor control, with EZH2-inhibited CAR T cells maintaining clearance for an average of 5.7 killing rounds compared with 14.0 rounds in untreated controls (174). Chronic EZH2 inhibition was also associated with loss of H3K27me3, increased expression of PD-1, CTLA-4, and LAG-3, and reduced CD8^+^ T-cell polyfunctionality, supporting a potential liability of sustained EZH2 inhibition within the engineered effector-cell compartment (174). This effect appears to depend strongly on treatment timing, because transient ex vivo tazemetostat exposure before the phenotypic onset of T-cell exhaustion increased TCF1 expression by reducing H3K27 methylation at the TCF7 (TCF1) promoter and preserving stem-like and polyfunctional T-cell features in preclinical models (140). These apparently divergent observations indicate that sustained systemic exposure and transient ex vivo epigenetic reprogramming should not be considered biologically equivalent, and they identify exposure duration and cellular compartment as important unresolved variables in designing EZH2 inhibitor–cell therapy combinations (140, 174). Tumor-intrinsic resistance can arise through distinct mechanisms. In SMARCB1-deficient epithelioid sarcoma and rhabdoid tumor models, genomic analyses of patient tumors together with functional studies identified acquired alterations converging on the RB1/E2F axis, allowing tumor cells to escape tazemetostat-induced G1 arrest despite continued EZH2 inhibition (182). These resistance-associated alterations uncoupled EZH2-dependent differentiation from cell-cycle control, providing a mechanistic example in which suppression of the intended epigenetic target persists but no longer produces the expected growth-arrest phenotype (182). Adaptive resistance may also involve broader changes in tumor cell state. In advanced prostate cancer models and human datasets, genetic or pharmacological EZH2 suppression expanded active transcription-factor programs and increased the diversity of lineage variants arising during disease progression, without preventing disease progression or therapeutic resistance (176). These findings suggest that, in tumors already capable of lineage plasticity, chromatin de-repression following EZH2 suppression may broaden the range of accessible cellular states rather than uniformly constrain tumor evolution (176). Longitudinal multi-omic profiling of cutaneous T-cell lymphoma similarly demonstrated substantial clonal evolution during progression and therapy resistance, including recurrent progression-associated alterations affecting EZH2 and multiple signaling pathways (177). However, this study (177) did not directly test resistance to pharmacological EZH2 inhibition; its relevance here is therefore to illustrate dynamic clonal and molecular evolution that may complicate static biomarker-based treatment strategies, rather than to establish EZH2 alteration as a causal mechanism of EZH2 inhibitor resistance.

Collectively, these findings define several non-equivalent routes to treatment failure, effector-cell impairment during prolonged drug exposure, tumor-cell escape from EZH2-dependent growth control, lineage-state diversification, and clonal evolution, and emphasize the need for longitudinal pharmacodynamic and molecular monitoring rather than reliance on baseline EZH2 expression alone. Whether these mechanisms can prospectively identify patients at risk of resistance or determine optimal dosing schedules and combination partners remains unresolved and requires validation in longitudinal clinical cohorts.

## 9 Future directions and emerging therapeutic paradigms

### 9.1 Matching the EZH2-targeting modality to biological dependency

A central priority for the next generation of EZH2-directed therapies is to move beyond a uniform “EZH2 inhibition” paradigm and determine which molecular function of EZH2 constitutes the relevant therapeutic dependency in each tumor context (24, 98, 117, 183). Where immune evasion is predominantly driven by PRC2-dependent H3K27me3 deposition, catalytic inhibition remains the most direct strategy. Conversely, tumors in which EZH2 protein abundance or methyltransferase-independent functions contribute to the malignant phenotype may require approaches that reduce the protein itself or disrupt specific non-catalytic interactions (24, 88, 98, 117, 183). Emerging protein degradation and post-translational strategies provide preclinical evidence that EZH2 can be targeted beyond its catalytic pockets. Peer-reviewed studies have described peptide-guided EZH2 degradation and modulation of EZH2 post-translational modifications (151, 167). However, additional small-molecule degrader activities, including that of AXT-1003, have only been reported in preliminary conference data (184). Collectively, these findings do not establish whether degradation or post-translational targeting is therapeutically superior to catalytic inhibition, particularly in immunocompetent tumor models or patients (151, 167, 184). Therefore, future studies should directly compare catalytic inhibition and EZH2 protein depletion within the same disease models while independently measuring total EZH2 protein, H3K27me3 target engagement, transcriptional reprogramming, and tumor- and immune cell phenotypes (24, 98, 117, 183, 184). Mechanistic discrimination also requires perturbation strategies that separate PRC2-dependent catalytic activity from EZH2 protein-dependent functions, including comparisons with other PRC2 components and appropriately designed EZH2 rescue experiments (24, 43–45, 117, 183). Such experiments will be necessary before non-catalytic or degradation-based approaches can be rationally assigned to specific tumor types rather than being deployed simply as more complete forms of EZH2 inhibition (24, 98, 117, 183). Translating these emerging modalities will also require rigorous evaluation of degrader selectivity, pharmacokinetics, tissue distribution, durability of EZH2 depletion, and effects on non-malignant immune compartments, as the removal of EZH2 protein may not phenocopy catalytic inhibition (29, 31, 123, 151, 184).

### 9.2 Precision deployment: compartment, timing, biomarkers, and rational combinations

A second priority is to determine not only how EZH2 should be targeted, but also where, when, and in which biological context EZH2 modulation should be applied (29, 123, 166, 175, 176). The opposing consequences of EZH2 perturbation across malignant cells, myeloid populations, and activated T cells argue against assuming that continuous systemic exposure will provide the optimal therapeutic configuration for every EZH2-based combination (29, 31, 123, 174). Accordingly, future therapeutic design should compare systemic treatment with temporally restricted, tumor-localized, and ex vivo approaches that seek to preserve tumor-directed epigenetic reprogramming while limiting unwanted exposure of immune-cell compartments (165, 166, 176). Nanoparticle and locally activated delivery systems provide preclinical proof of concept that EZH2 modulation can be concentrated within the tumor treatment field, but these platforms should not yet be regarded as genuinely cell-type-selective; their biodistribution, intratumoral heterogeneity, reproducibility, and safety require systematic evaluation (165, 166).

Transient ex vivo EZH2 modulation represents a conceptually distinct strategy from prolonged systemic inhibition and should be optimized separately with respect to exposure duration, differentiation state, persistence, and effector function (140, 141, 174). An additional proof-of-concept approach is the engineering of EZH2 inhibitor-resistant CAR T cells to uncouple tumor-directed EZH2 inhibition from T-cell-intrinsic drug exposure; however, this strategy remains preliminary and conference-reported and requires independent validation before its translational feasibility can be assessed (175). Rational combination therapy should likewise be selected according to the dominant immune-evasion bottleneck rather than by empirically adding EZH2 inhibition to immune therapy across unselected tumors (88, 92, 93, 100).

For example, tumors with reversible epigenetic loss of antigen presentation provide a mechanistic rationale for pairing EZH2 targeting with checkpoint blockade, whereas tumors with suppressed endogenous innate-sensing pathways may require combinations that restore viral mimicry or STING-dependent signaling (88, 92, 93, 100). These pathway-matched combinations should be contingent on demonstrating that the downstream machinery required for the proposed immune response remains functional, because epigenetic de-repression alone cannot compensate for irreversible defects in the relevant recognition or signaling pathway (88, 92, 93, 100). Therefore, patient selection should progress beyond EZH2 transcript or protein abundance toward composite biomarkers that incorporate functional EZH2/PRC2 activity, H3K27me3 target engagement, antigen-presentation competence, interferon or innate-sensing capacity, and the composition and spatial organization of the tumor immune microenvironment (85, 88, 93). Spatial and single-cell profiling may be particularly useful for identifying tumors in which a potentially favorable tumor-cell response to EZH2 targeting coexists with myeloid or lymphoid compartments that could be adversely affected by the same intervention (29, 31, 123). Consequently, prospective trials should incorporate paired or longitudinal pharmacodynamic sampling wherever feasible, enabling the evaluation of target engagement, immune-state transitions, and emerging clonal or lineage changes, rather than relying exclusively on baseline EZH2 expression (85, 86, 176, 177). Therefore, the broader objective is not universal EZH2 inhibition but mechanism-matched, compartment-aware, and temporally controlled EZH2 modulation, with the targeting modality, combination partner, treatment schedule, and biomarker strategy selected according to the dominant resistance mechanism in each disease context (29, 31, 85, 86, 123, 140, 176).

### 9.3 Cancer prevention, early interception, and durable immune control

A conceptually distinct and currently hypothesized dimension of EZH2 biology concerns whether the same epigenetic circuits that govern immune recognition in established tumors may also operate at earlier stages of malignant transformation, raising the possibility that early immune modulation can be examined in premalignant conditions. In oral leukoplakia, EZH2 expression in the epithelium has been reported to correlate positively with CD3^+^ and CD8^+^ T cell infiltration and is associated with a substantially elevated risk of transformation to oral squamous cell carcinoma during follow-up (81). This counterintuitive coupling of elevated EZH2 with abundant but apparently non-protective T cells raises the possibility that immune invisibility at the premalignant stage may be established by functional disarmament rather than numerical exclusion. Mechanistically, single-plex and multiplex immunohistochemistry, single-cell sequencing, and CUT&RUN analyses in oral potentially malignant disorders have associated EZH2 with H3K27me3 deposition on *HLA-A/B/C*, *B2M*, and *TAP1* promoters, reduced antigen-processing machinery expression, and diminished CD8^+^ GZMB^+^ infiltrates (105). A mucoadhesive hydrogel loaded with GSK126 (PPT@GSK126) has been explored as a locally delivered investigational tool, and in preclinical systems, it has been reported to be associated with restored MHC-I expression and, in combination with topical anti-PD-1, with reduced progression of these lesions (105). However, these observations were derived from animal models and do not constitute evidence for clinical prevention. A separate preclinical study in a Lynch syndrome model reported that pharmacological EZH2 inhibition reduced H3K27 methylation, increased immune cell representation in relevant lymphoid and colonic compartments, and reduced intestinal adenoma multiplicity, extending the immune-interception hypothesis to mismatch repair-deficient colorectal tumorigenesis while remaining non-clinical evidence (185). These interception concepts must, however, be reconciled with the safety signal described in Section 8.1. Because prevention and interception require prolonged exposure in individuals without established malignancy, the observation of hematologic second primary malignancies during extended tazemetostat administration sets a substantially higher safety threshold for this indication than for advanced disease, and supports locally delivered or otherwise compartment-restricted formulations rather than systemic dosing as the only currently defensible route for further preclinical development.

A related concept concerns durable immune control at later stages through modulation of the SASP. In pancreatic ductal adenocarcinoma (PDAC) models, EZH2 has been reported to epigenetically repress proinflammatory SASP genes (*CCL2*, *CXCL9/10*) following therapy-induced senescence, and EZH2 blockade has been associated with NK and T cell infiltration (118). These findings have been framed within an immunoediting perspective, in which EZH2 may contribute to immune escape across multiple disease stages (23).

Current evidence is derived from retrospective correlative cohorts and murine/hydrogel preclinical systems with limited longitudinal human data. Premalignant lesions are biologically heterogeneous, the feasibility of sustained early intervention and the safety of long-term EZH2 modulation are undefined, and no prospective prevention or interception trial exists. Collectively, these observations may provide a conceptual basis for an interception hypothesis but require prospective validation, and cancer prevention remains to be determined.

## 10 Conclusion

EZH2 is best viewed as a context-dependent regulator at the epigenetic–immune interface, linking tumor-intrinsic chromatin programs that influence immune recognition with the functional state of immune cells in the tumor microenvironment. The immune consequences of EZH2 perturbation depend on the tumor context, cellular compartment exposed, targeting modality, and treatment duration, with favorable tumor-directed effects potentially offset by adverse effects in myeloid or activated T-cell compartments. Although early-phase human studies establish the feasibility of combining tazemetostat with PD-1/PD-L1 blockade or CAR T-cell therapy, controlled evidence demonstrating incremental clinical benefit from EZH2 inhibition remains lacking. Future development should therefore prioritize functional biomarkers, longitudinal pharmacodynamic assessment, and compartment- and schedule-aware therapeutic strategies rather than universal EZH2 inhibition.
